# Solving the Large-Scale TSP Problem in 1 h: Santa Claus Challenge 2020

**DOI:** 10.3389/frobt.2021.689908

**Published:** 2021-10-04

**Authors:** Radu Mariescu-Istodor, Pasi Fränti

**Affiliations:** School of Computing, University of Eastern Finland, Joensuu, Finland

**Keywords:** GRID, graphs, clustering, divide and conquer, TSP, scalability

## Abstract

The scalability of traveling salesperson problem (TSP) algorithms for handling large-scale problem instances has been an open problem for a long time. We arranged a so-called Santa Claus challenge and invited people to submit their algorithms to solve a TSP problem instance that is larger than 1 M nodes given only 1 h of computing time. In this article, we analyze the results and show which design choices are decisive in providing the best solution to the problem with the given constraints. There were three valid submissions, all based on local search, including *k*-opt up to *k* = 5. The most important design choice turned out to be the localization of the operator using a neighborhood graph. The divide-and-merge strategy suffers a 2% loss of quality. However, via parallelization, the result can be obtained within less than 2 min, which can make a key difference in real-life applications.

## Introduction

The traveling salesperson problem (TSP) (Applegate et al., 2011) is a classical optimization problem that aims to find the shortest path that connects a given number of cities. Even though the name implies outdoor movement by human beings, there are many other applications of TSP, including planning, scheduling of calls, manufacturing of microchips, routing of trucks, and parcel delivery.

TSP is not limited to physical distances, but any other optimization function in place of the distance can be used. In the early days, travel distance or travel time were used (Dantzig and Ramser, 1959), but currently, other objectives also exist, such as minimizing the exposure to sunlight (Li et al., 2019), maximizing the safety of travel (Krumm and Horvitz, 2017), or minimizing CO_2_ levels (Kuo and Wang, 2011; Lin et al., 2014). Practical applications include logistics such as vacation planning or orienteering (Fränti et al., 2017), transportation of goods, providing maintenance, or offering health services (Golden et al., 2008), among others.

Sometimes there is a need to process larger problem instances, especially in applications such as waste collection and delivery of goods, where millions of deliveries occur each day. Due to the COVID-19 pandemic, logistics became the center of attention, causing couriers to struggle to meet increased demands from hospitals, supermarkets, and those who self-isolate at home and order food and merchandise online.

TSP is an NP-hard problem. Despite this fact, there are algorithms that can solve impressively large instances. Some of these are exact solvers that guarantee the optimum solution (Barnhart et al., 1998; Applegate et al., 1999; Bixby, 2007). However, these methods have exponential time complexity and become impractical on large-scale problem instances. The current record includes 85,900 targets that were solved in approximately 136 CPU-years (Applegate et al., 2011). For this reason, heuristic solvers have been developed, and they currently outnumber the exact solution methods.

Existing heuristics use different strategies, such as local search, tabu search, ant colony optimization, simulated annealing, and evolutionary methods (Braekers et al., 2016). These methods can quickly provide suboptimal solutions that are acceptable in practice. For example, we can solve an instance with 1,904,711 targets within 15% of the optimum in 30 min and within 0.1% of the optimum in a few hours (Helsgaun, 2000; Taillard and Helsgaun, 2019). The quality of the solution increases with the processing time the algorithm spends.

A generalized variant of TSP is the vehicle routing problem (VRP) (Dantzig and Ramser, 1959). This problem considers several real-world elements, such as multiple agents (*k*-TSP), limited capacity, working hours, time windows, and depots. VRP instances are more difficult to solve, and most research considers instances with only a few hundred targets (Dumas et al., 1991; Reinelt, 1992; Uchoa et al., 2017). In fact, the first instances with up to 30,000 targets appeared just last year (Arnold et al., 2019), even though there is a real demand for even much higher instances. FedEx, for example, have over six million deliveries per day.^1^

When solving large-scale problem instances, the scalability of the algorithms is still an issue. One issue is that the many algorithms are not linear in time and therefore not scalable. Another issue is that many implementations use a complete distance matrix that cannot fit into memory with large-scale instances. One solution is to split the problem into subproblems and use multiple cores. However, NP-hard problems cannot be naturally divided without compromising the quality. It is not trivial to create a good split-and-merge strategy that would keep the loss of quality marginal. All of these issues pose challenges to applications of TSP to large-scale problem instances.

The concept of large-scale itself has evolved considerably over the years. In a study by Thibaut and Jaszkiewicz (2010), problem sizes up to *N* = 1,000 were considered. Sakurai et al. (2006) performed experiments with only *N* = 40 targets, and the problem instances of *N* = 1,000–10,000 were called very large scale. One obvious approach to attack the issue is to divide the problem into smaller subproblems. Different space partitioning methods have been used, such as Karp, Strip, and Wedging insertion (Valenzuela and Jones, 1995; Xiang et al., 2015). The affinity propagation clustering algorithm was used by Jiang et al. (2014) for problems of size *N* < 3,000 and hierarchical k-means for problems of size *N* > 3,000. Each cluster had 40 targets at the most. However, the reported results took 3 days to compute problem sizes over 1 M.

When using clustering, it is also possible that some clusters are larger than others, which could lose the benefit of clustering. An attempt to reach balanced cluster sizes was considered by Yang et al. (2009). Another problem is memory consumption because many algorithms store an *N*×*N* distance matrix. Additional memory is needed in some cases, such as storing the pheromone trails in the case of ant colony optimization. Nevertheless, a version of ant colony optimization by Chitty (2017) was capable of handling these issues and solving problem sizes up to 200 k at the cost of a 7% decrease in accuracy. This cost (decrease) in the performance is quite large to pay for the scalability.

To address this problem, we created a TSP challenge where participants were asked to provide an algorithm to optimize Santa Claus tours to visit all households in Finland. The algorithms should terminate within 1 h. In this article, we perform an analytical comparison of the submitted algorithms. We analyze each design component separately, including the single-solution TSP solver, clustering method for dividing the problem into subproblems, merge strategy to combine the subsolutions, approaches to the open-loop, fixed start point, and *k*-TSP cases.

### Data and Goals

In the spirit of Christmas, Santa Claus needs to deliver presents to the children in every family on Christmas Eve. He can also use *k* helpers by dividing the tour into *k* parts accordingly. We awaited solutions to the following problem cases:

1. Closed-loop TSP;
2. Open-loop TSP;
3. Fixed-start TSP (open loop);
4. Multiple tours *k*-TSP (open loop).

The first case is the classical (closed-loop) TSP problem where Santa needs to return home to complete the tour. The three other cases are open-loop variants that create a TSP path where return to home is not necessary. In the second case, Santa can start from any location with the logic that he has plenty of time to prepare and can go to the selected start point well before the actual trip starts; only the time spent traveling the path counts. In the third case, he leaves from his home (depot), which in our data is set to Rovaniemi. The fourth case is motivated by the fact that it would be impossible for Santa to make the trip on Christmas Eve without breaking the laws of physics.^2^ Santa therefore recruits *k* assistants; elves or drones in modern times (Poikonen et al., 2019) and divides the tour into multiple parts that are solved by each helper independently.

### Data

We extracted all building locations in Finland from OpenStreetMap^3^ (OSM) to create 1,437,195 targets that represent the households for Santa to visit. We used our local installation of the OSM and the Overpass API. We first queried inside a bounding box that encompasses the entire country and kept only the buildings that fell within the country borders. The coordinates were converted into the Finnish National Coordinate System (KKJ), which projects them in Euclidean space (most typical for researchers), where the Euclidean distance corresponds to meters of movement in a straight line. The data are published as a TSP instance on our website.^4^

**FIGURE 1:**
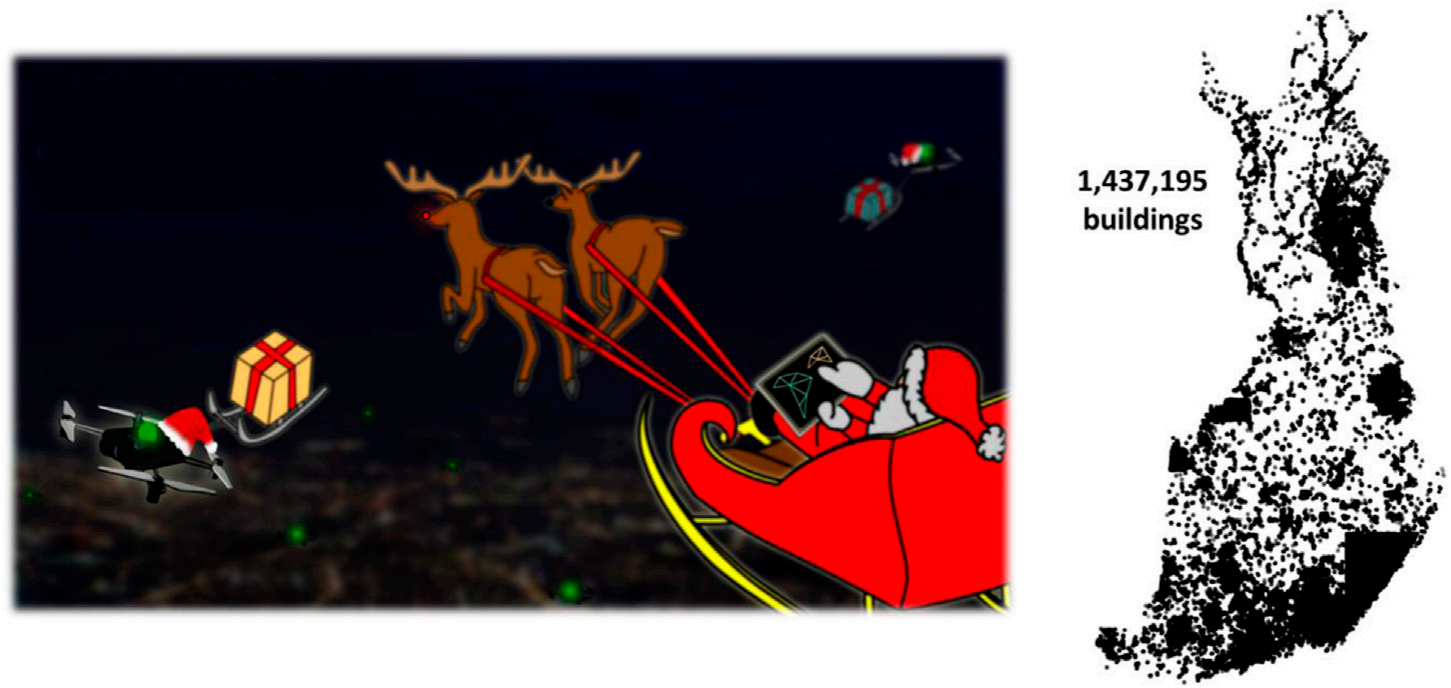
The Santa Claus TSP challenge was motivated by Santa's need to deliver presents to the children in every family on Christmas Eve, as is the tradition in Finland. We used publicly available data from OSM, consisting of 1.4 million building locations. While most of the population is in south Finland, this data set has dense coverage of certain regions, such as southeast Finland. We note that this database does not cover the entirety of Finland, and there is a bias in the locations.

We note that the building locations do not match 1:1 to the households in Finland, and there is bias. Some regions, especially in the southeastern part, have denser records of buildings than the other areas. This arrangement shows visible artifacts as sharp edges between the dense and sparse regions (see Figure 1). This appearance is most likely due to an inconsistent representation in the OSM database of north vs. south. The data itself appear to be correct, but there is a lack of data in many regions. However, for the purpose of our Santa competition, the resulting data set is good enough, because the size of the data serves the need for a large-scale test case (*n* ≈ 1.4 M).

### Goals

We set a requirement that a submitted algorithm must optimize the tour within 1 h. All of the valid submissions were evaluated after executing for 1 h or less. A program was disqualified if it did not terminate within 1 h. All of the submissions were evaluated in terms of quality, speed, and simplicity. We ran all of the algorithms on the same Dell R920 machine with 4 × E7-4860 (a total of 48 cores), 1 TB, and 4 TB SAS HD.

While Santa has time during the entire year for his preparations, we set a stricter requirement. We allowed the computer program to spend only 1 h performing the necessary calculations. This approach reflects more real-life applications where the situation is dynamic and constantly changing. The purpose is to test the scalability of algorithms for real-life applications.

### Challenges

The greatest challenge is the size of the data and the limited processing time of 1 h. Most TSP solvers have quadratic or higher time complexity, and even simple greedy heuristics need to compute the distance to all remaining targets at each of the *n* steps (Fränti et al., 2021). Even such algorithms are not fast enough to complete in 1 h on the specified machine.

Another challenge is the multiple variants. Most existing methods are tailored for the classical closed-loop case, and some modifications are needed to adapt them to the open-loop, fixed-start, and *k*-TSP cases.

## Solving Large-Scale Problems

The general structure of all methods discussed here follows the same overall structure, which consists of the following components:

• Single-solution TSP solver,
• Divide into smaller subproblems,
• Merge the subsolutions.

### Single-Solution TSP Solver

State-of-the-art TSP solvers (excluding the optimal ones) and all submitted algorithms are based on local search. The idea is to have an initial solution that is then improved by a sequence of local operators in a trial-and-error manner. The key component in the local search is the choice of the operator. It defines how the current solution is modified to generate new candidate solutions. The following operators were used:

• Relocate (Gendreau et al., 1992),
• Link swap (Sengupta et al., 2019),
• 2-opt (Croes, 1958),
• *k*-opt (Shen and Kernighan, 1973).

**FIGURE 2:**
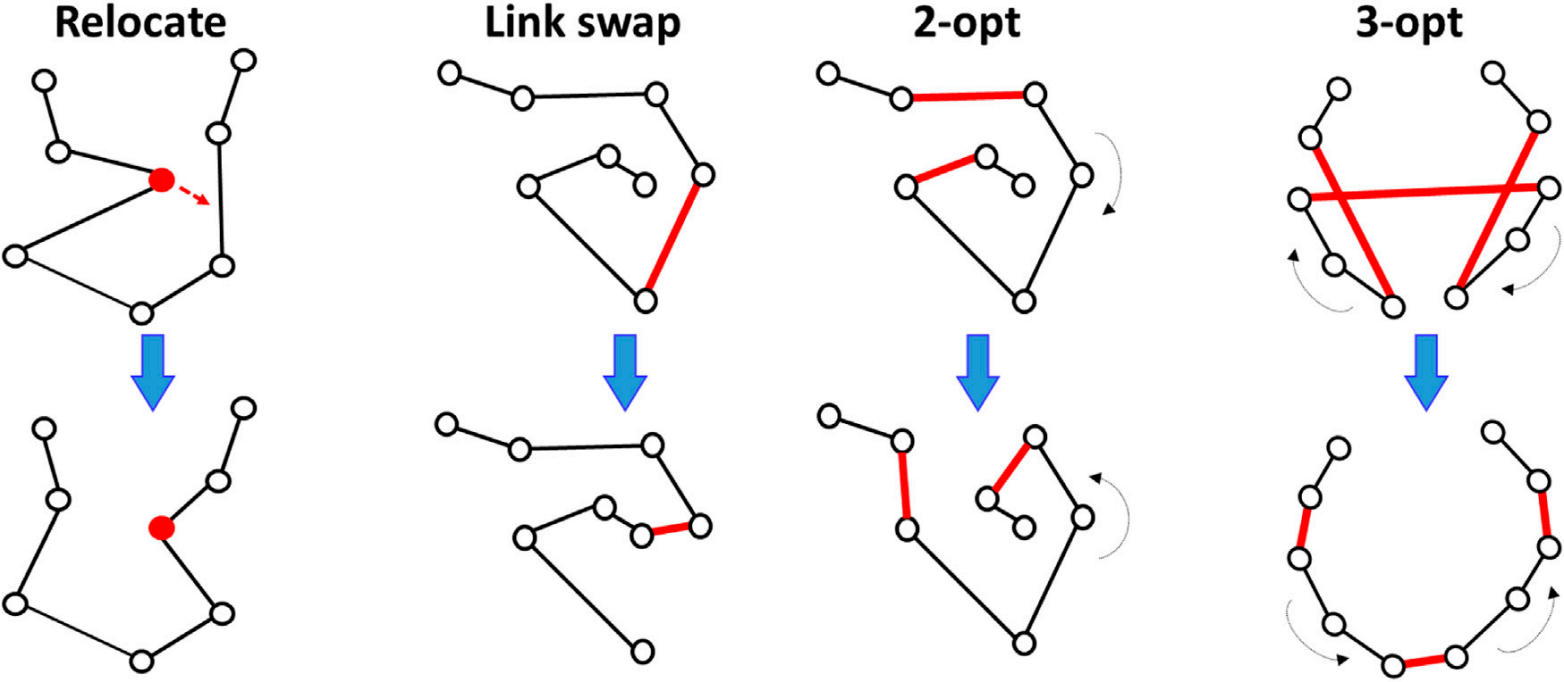
Local search operators used in the submitted algorithms.

The most popular of these is 2-opt (Croes, 1958) and its generalized variant *k*-opt (Shen and Kernighan, 1973). The 2-opt operator selects two links and redirects the links between their nodes. Its generalized variant, *k*-opt, involves *k* links, which allows more complex modifications of the solution. It is also known as the Lin–Kernighan heuristic. A Link swap relocates any link by connecting it to one or both end points of the current path. Link swap works only for the open-loop case but has been found to contribute most to the search; approximately 50% of the improvements were due to Link swap as shown by Sengupta et al. (2019). Relocate removes a node from its current position in the path and reallocates it elsewhere by creating a new detour via this node. The operators are demonstrated in Figure 2. Tilo Strutz pointed out that both Relocate and Link swap are special cases of *k*-opt (Strutz, 2021).

Most local search algorithms apply one of the three strategies: random, best improvement, and prioritization. Random search tries out the operators in random order and accepts any solution that improves the current solution. Best improvement considers all possible operators for the current solution and selects the best. The operators and their parameters can also be prioritized based on criteria such as alpha-nearness in an effective implementation of the Lin–Kernighan heuristic known as LKH (Helsgaun, 2000). Here, each link is scored based on sensitivity analysis using minimum spanning trees (MST). More specifically, it measures the increase in cost when a tree is forced to contain the specific link.

### Localizing the Search Space

**FIGURE 3:**
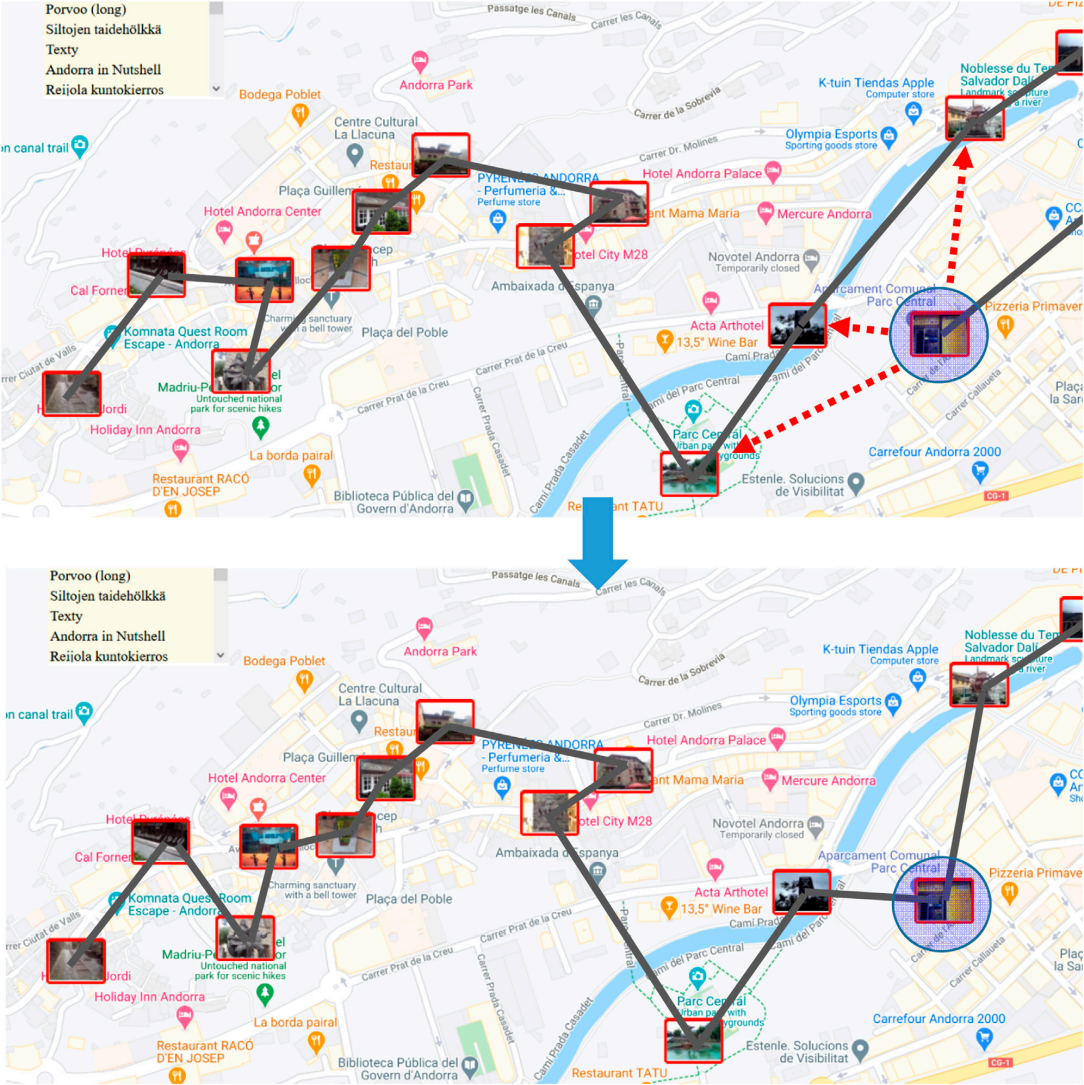
Local search operators should consider only nearby points and links. A given target has *n* − 2 = 13 new positions, where it could be relocated. However, only the nearby points (marked by arrows) are worthwhile considering.

One limitation of the above-mentioned operators is that most of the generated candidates are meaningless. For example, it makes no sense to try to relocate a node along a path at the opposite part of the space. In most cases, only the nearby points are meaningful (see Figure 3). In the case of relocation and 2-opt, only a few out of the *n* − 2 choices have a realistic chance of success, and most others would be just a waste of time. Link swap is somewhat better because there are only three choices, of which at least the one where both end points are changed is potentially meaningful.

**FIGURE 4:**
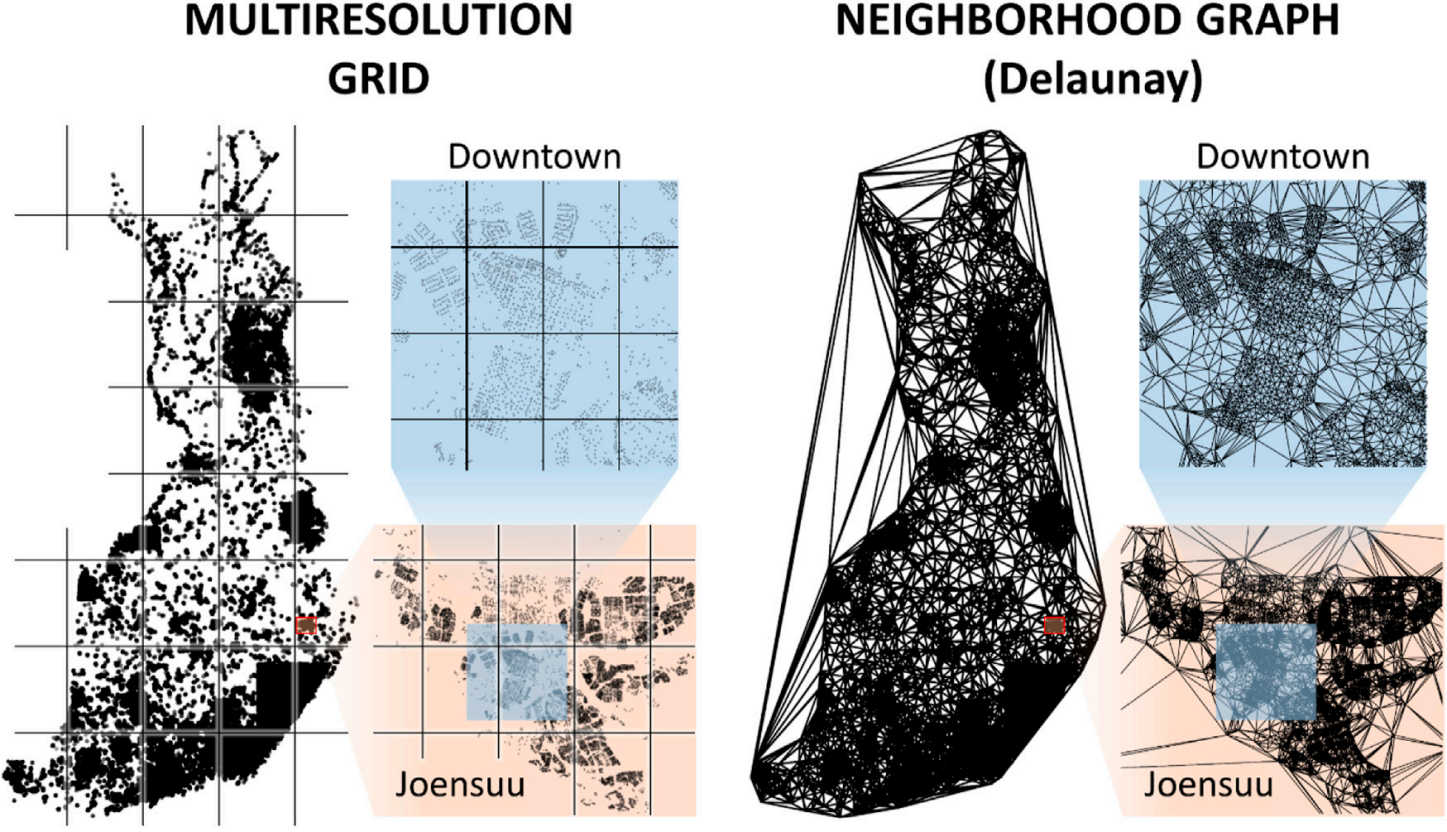
Efficiency of the local search operators depends highly on how to limit the search for candidates within the neighborhood. The grid-based approach restricts the two operands (links) to be in the same or neighboring grid cells, while a neighborhood graph considers only connected nodes.

Two strategies have been applied to restrict the operators to consider targets only in the local neighborhood: grid and neighborhood graph. The first strategy is to divide the space into cells by generating a grid (see Figure 4). The operator is then restricted to considering only nodes and links within the same cell or targets in neighboring cells. Because the density of data varies considerably and the goal is to limit the number of nodes per cell, multiple resolutions are often necessary, and smaller cells are generated only when needed.

The second strategy is to create a neighborhood graph such as the Delaunay graph or related graphs such as Gabriel or XNN. Efficient algorithms exist to compute these data structures for 2D geographic data; a linear time solution for the Delaunay graph can be found in the work by Peterson (1998).

The drawback of localization is that successful operations can be missed due to arbitrary grid divisions or limitations in the neighborhood graphs. However, it was shown by Fränti et al. (2016) that 97% of the links in the optimal TSP path are included in the XNN graph. Localization is an efficient approach to reduce unnecessary calculations and is expected not to miss many of the moves that full search would potentially find.

### Divide by Clustering

To make the algorithm scalable, we expect that it is necessary to divide the data into smaller size instances that the TSP solver can manage. Spatial clustering is applied here. The idea is that each subproblem is solved separately, and the resulting paths are then merged to create the overall TSP path.

To select the most appropriate clustering method, we must consider three questions: 1) which objective function to optimize, 2) which algorithm to perform this optimization, and 3) how many clusters. Assume that the TSP solver requires quadratic, O(*N* ^2^), time complexity. If the data are equally divided into √N clusters, we would have √N points in each cluster. Solving TSP for one cluster would require O(*N*) time and O(*N* ^1.5^) for all √N clusters. With our data (≈1.4 M points), we would have approximately 1,200 clusters. Multithreading with >1,200 processors would make the computation time linear.

**FIGURE 5:**
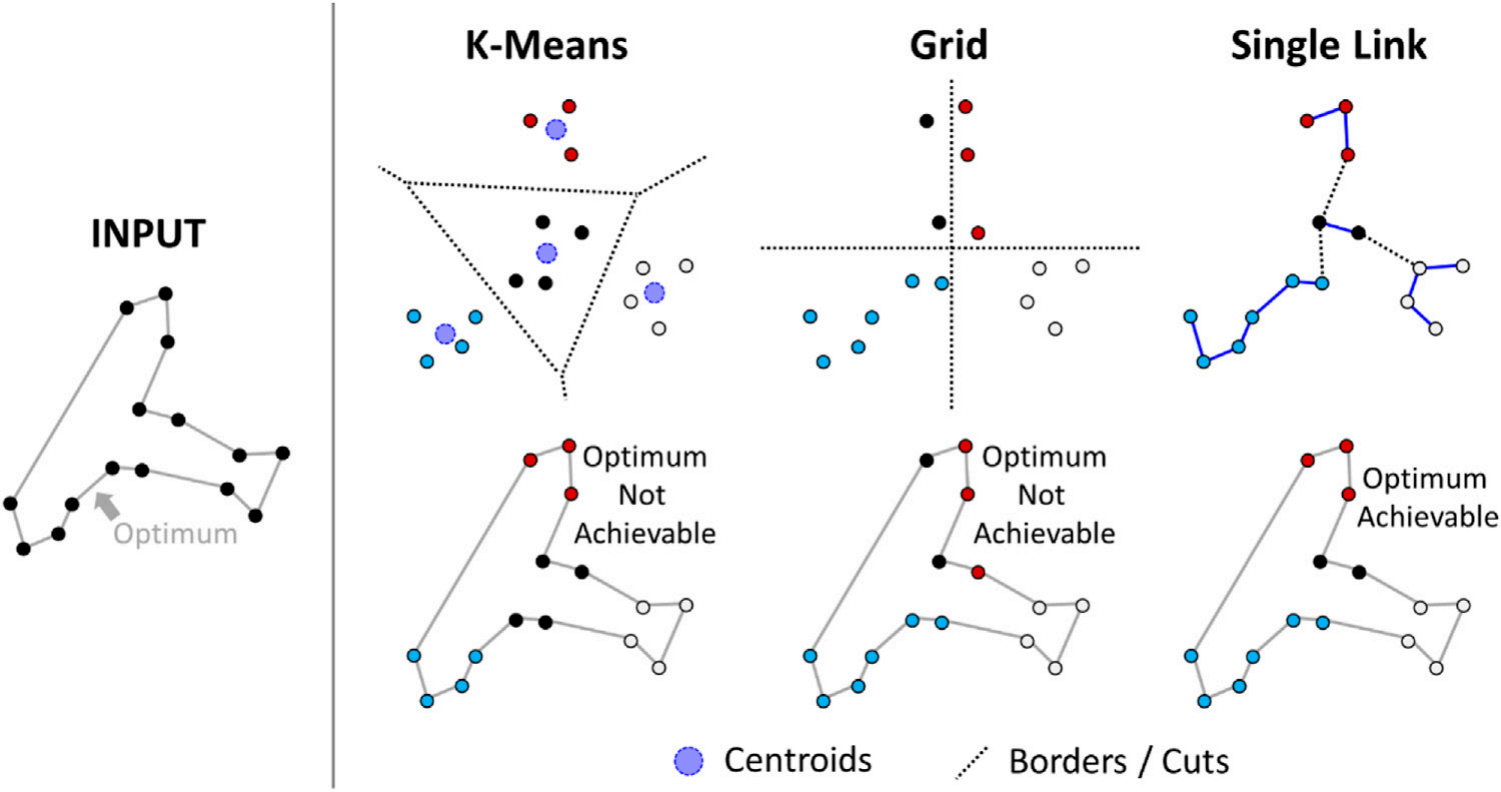
An example of a TSP instance divided into clusters in three different ways. The optimum cannot be achieved when it enters the same cluster more than once.

However, it is not clear how the clusters should be optimized. Figure 5 shows several possible methods. K-means minimizes the sum-of-squared errors and generates spherical clusters. This approach is a reasonable clustering strategy in general but not necessarily the optimal strategy for TSP. Grid-based methods are faster but even worse, in general, because the borders are decided without considering any properties of the data. On the other hand, the TSP path is also a spanning tree, and the clusters correspond to a spanning forest. A single-link clustering algorithm might therefore make sense because it finds a minimum spanning forest. However, when the density varies, large clusters will form in regions with high density (southern Finland), which are too large to be optimized properly. Attempts to mitigate this behavior were tried out by Mariescu-Istodor et al. (2021), but those approaches were too slow to handle more than a few tens of thousands of targets efficiently. The relationship between MST and TSP was also utilized by Fränti et al. (2021). Density peaks clustering was considered by Liao and Liu (2018).

### Merging the Subsolutions

Another open question in the algorithms is how to merge the subtours from the individual clusters into a single tour. All divide and conquer submissions decide the merging strategy before solving the clusters. The problem is essentially formulated as a cluster-level TSP, where each cluster represents a node in a so-called meta-level graph. This meta-level problem is solved to decide which clusters are to be merged (Kobayashi, 1998). The subproblems within each cluster are treated as open-loop TSP instances with fixed end points, which are used for connecting the clusters.

The divide-and-conquer approach optimizes the subsolutions and the overall tour independently. It is likely to lead to some suboptimal choices, which makes it also possible to improve the overall tour later by applying additional operators as a fine-tuning step. This outcome can significantly improve the solution, especially across the cluster boundaries. Here, the same single-solution TSP solver can be applied, but since the entire problem instance is much larger, this approach can be quite time consuming and can be applied only by a limited number of iterations. Localization of the operations is therefore necessary in this fine-tuning step.

### Solutions From the Literature

In the literature, Karp (1977) applied a divide-and-conquer technique with a k-d tree-like structure in 1997. Two heuristics (including Lin and Kernighan) and one optimal heuristic (dynamic programming) were considered for solving the subproblems. Problem sizes of *N* = 128 were used. No TSP experiments were provided, but gap values of the corresponding MST solutions using 16 clusters varied from 3.7 to 10.7%.

Valenzuela and Jones (1995) extended Karp's idea by selecting the direction of a cut via a genetic algorithm and by introducing a method to merge the subsolutions. The results within a 1% gap to the optimal were reported at the cost of 12–28 h of processing time for a problem instance of size *N* = 5,000. Parallel implementation of Karp's algorithm was described by Cesari (1996), and processing times less than 1 h were reported using 16 processors for problem sizes up to *N* = 7,937. Modest gap values (approximately 5%) were reported with only two subproblems.

Things started to develop significantly in 2003 when Mulder and Wunsch (2003) applied the adaptive resonance theory algorithm of Carpenter and Grossberg (1998) to cluster data by a hierarchical k-means variant in combination with a variation of the Lin–Kernighan heuristic. It reached an 11% gap with a problem size of *N* = 1 M in which there were randomly distributed cities, and the solution spent only 16% of the time required by the full version (24 min vs. 2 h 30 min). In the same year, 2003, the WorldTSP^5^ data set was published, which consisted of 1.9 M city locations. In 2020, Geonames^6^ was published with 12 M geographical features on Earth. Also in 2020, GalaxyTSP was published (Drori et al., 2020) and extended the largest size ever considered by containing 1.69 billion stars.

A quadtree-based divide-and-conquer technique was applied by Drori et al. (2020) using LKH to solve the subproblems in parallel. It took 50 min to solve the WorldTSP, reaching a 1% gap compared to the best known result. The current record holder is Keld Helsgaun (15th Feb. 2021), who used essentially the same LKH method that was submitted here to the Santa competition. To solve the GalaryTSP, the data were divided into 1,963 tiles with an average size of 861,913 nodes, and it took approximately 3 months to solve the problem in parallel (Drori et al., 2020).

Overall, the state of the art in the literature still appears to rely mainly on local search and LKH. A variant of the LKH was compared against a genetic algorithm with an edge assembly crossover by Paul et al. (2019) to determine which method finds the optimal solution faster given a 1-h time limit. The genetic algorithm was better in 73% of the cases, but only problem sizes up to *N* = 5,000 were considered. However, optimality was required, and the authors expected the processing times to align when the problem size increased. We cannot therefore conclude much about how the genetic algorithm would perform with the Santa competition, in which the success is measured by the gap value.

### Other Cases

**FIGURE 6:**
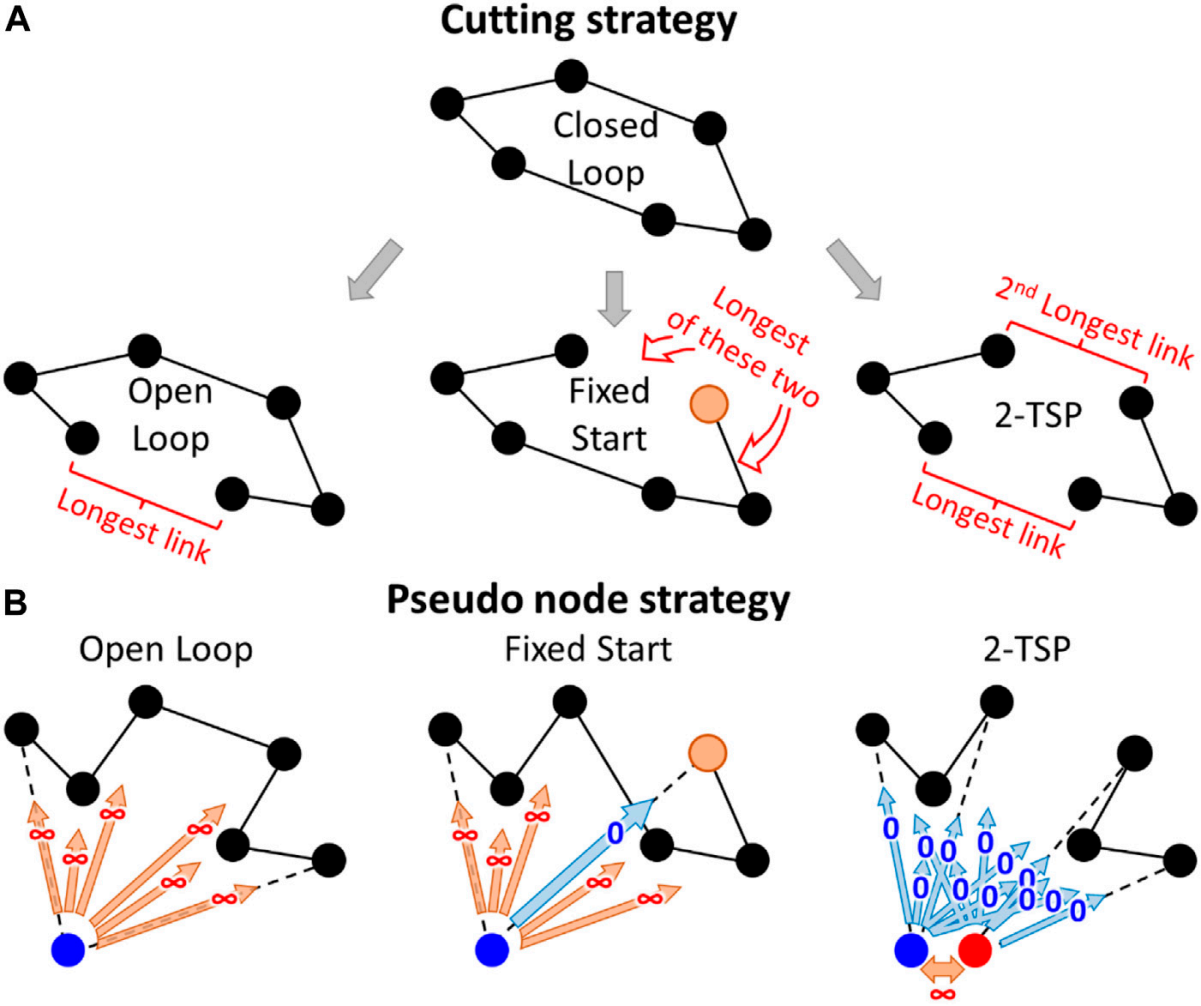
A straightforward approach **(A)** and the pseudocode approach **(B)** to generate open-loop, fixed-start, and *k*-TSP (*k* = 2) solutions from the closed-loop solution.

A straightforward approach to support several TSP variants is to create a base algorithm that solves the closed-loop TSP and then to make small modifications to the result to generate solutions for the open-loop, fixed-start, and *k*-TSP cases. For example, an open-loop solution (TSP path) can be created by simply cutting the longest link from the closed-loop variant (TSP tour). A fixed start point can also be created in the same manner by cutting one of the links connected to Santa's home. The same idea generalizes to the *k*-TSP case by removing *k* links (see Figure 6). However, this variant is slightly naïve. For example, removing the longest link from the optimal closed-loop solution does not necessarily create an optimal open-loop solution.

A more sophisticated approach is to create a pseudonode that has a constant distance to all other nodes (Sengupta et al., 2019). Because of the equal distances, it does not matter which nodes it will be connected to. However, it allows the remainder of the tour to be optimized as a path and to avoid one long link somewhere along the tour. The two nodes connected to the pseudonode will represent the start and end points of the open-loop variant. The chosen distance to the pseudonode does not matter if the TSP solver is optimal. However, it was noted by Fränti et al. (2021) that it is better to use some large constant, whereas using zero values works poorly with some heuristics. A fixed start point can be dealt with by setting its distance to the pseudonode to 0. This approach will avoid one infinite link and guarantees that the selected node will become one of the end nodes in the TSP path.

## Submitted Methods

In total, 82 trial TSP solutions were submitted to the testing website by 13 different authors to see how they ranked. However, only four algorithms were eventually submitted to the competition:

• Keld Helsgaun,
• Tilo Strutz,
• UEF,
• Anonymous.

Three worked as expected. They all were based on local search. The fourth failed to work even if it ran significantly longer than the time limit. Originally, we did not plan to submit our own method because we were the organizers of the event. However, since the number of submissions remained small, we decided to contribute ourselves by using our simple baseline solver from Sengupta et al. (2019) with the clustering-based divide-and-conquer approach that we had implemented earlier to serve as a reference solution. We did not expect it to perform well in this competition.

### Keld Helsgaun

**FIGURE 7:**
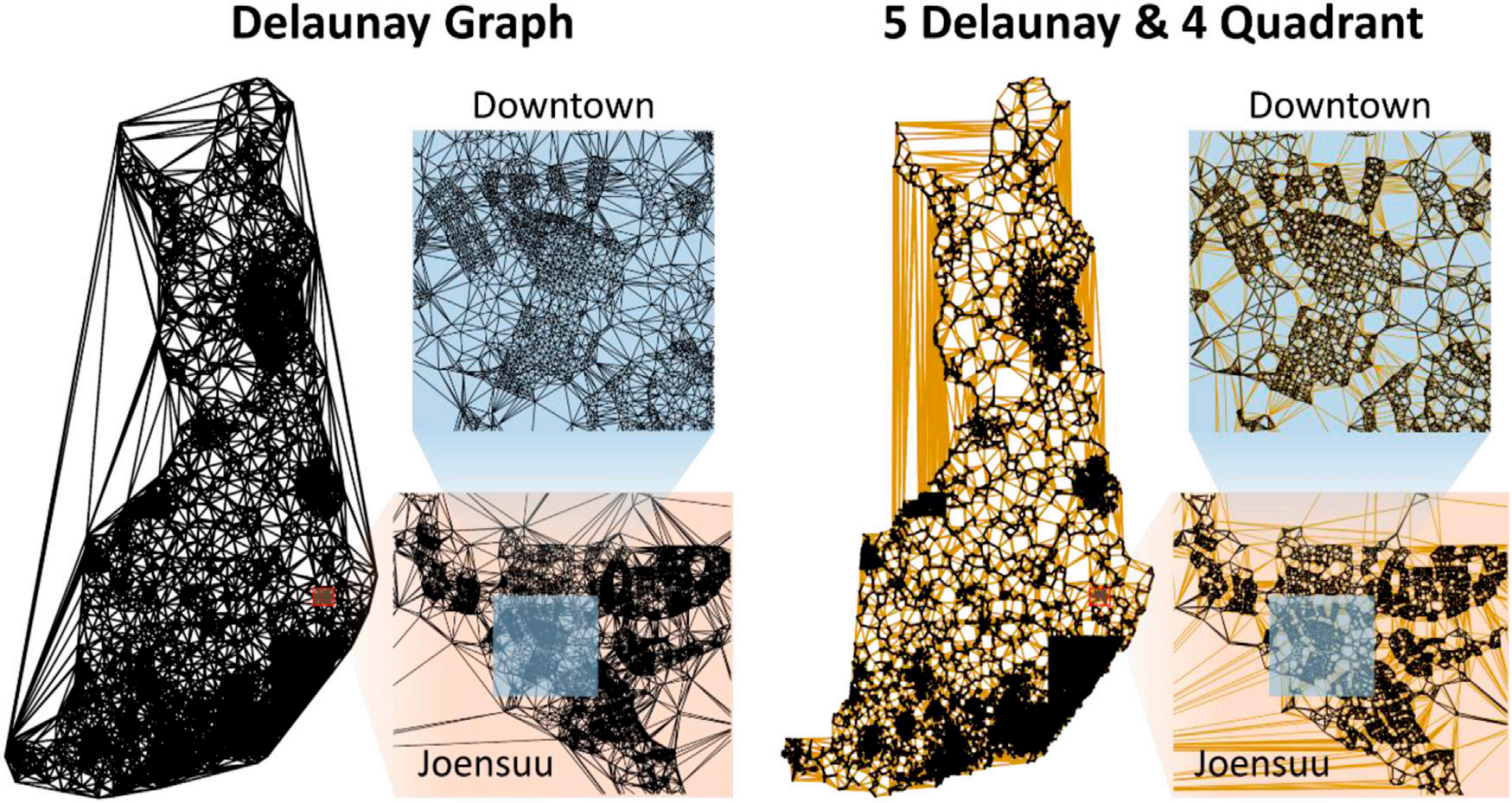
Comparison of Delaunay links and those used in the LKH submission.

Keld Helsgaun submitted his algorithm known as LKH^7^ with minor modifications and with specific parameter settings. It is an efficient implementation of the LKH that supports up to *k* = 5. The algorithm uses a best-improvement strategy, because it tries all combinations and selects the best combination when updating the tours. This process is usually slow, but the author modified it to have much better efficiency by precomputing a candidate set of links to be considered instead of trying all possible links. This strategy is accomplished by building a graph that preserves the neighborhood, such as using Delaunay triangulation (Reinelt, 1992; Peterson, 1998). LKH supports several options, of which Keld Helsgaun considered a hybrid of Delaunay and Quadrant links when building the graph (see Figure 7). The quadrant graph is formed using the nearest neighbors in each of the four quadrants centered at a given point.

**FIGURE 8:**
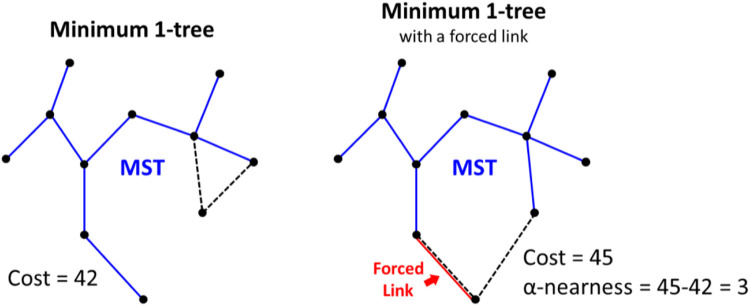
An example of how alpha nearness is calculated for a given link with respect to the minimum 1-tree and the 1-tree that includes the forced link.

The resulting links are prioritized using alpha-nearness: the increase in cost when a minimum 1-tree is required to contain the link to be scored (see Figure 8), where a 1-tree is a spanning tree defined on a node set minus one special node combined with two links from that special node. A 1-tree is actually not a tree because it contains a cycle. LKH is primarily designed for the closed-loop variant, where the 1-tree concept is especially meaningful; a TSP tour is a 1-tree where all nodes have a branching factor of 2. The optimum TSP tour is the minimum 1-tree with all branching factors equal to 2.

Computing all alpha-nearness values is time consuming O(*N* ^2^) and will complete in several days on an instance the size of Santa data. Fortunately, approximation is possible using subgradient optimization (Held and Karp, 1971). This process has been shown to eventually converge to the true alpha values, but stopping it early is the key to high efficiency. This goal is accomplished in LKH by setting a small initial period for the ascent (INITIAL_PERIOD = 100, default is *N*/2).

A third necessary parameter setting is TIME_LIMIT. It is set to 3,000 s (50 min) because time starts ticking only after the preprocessing is done: generating the candidate sets and estimating the alpha-nearness values take approximately 10 min.

The other parameters were the following:

INITIAL_TOUR_ALGORITHM = GREEDY

MAX_SWAPS = 1,000 maximum number of swaps allowed in any search for a tour improvement

**FIGURE 9:**
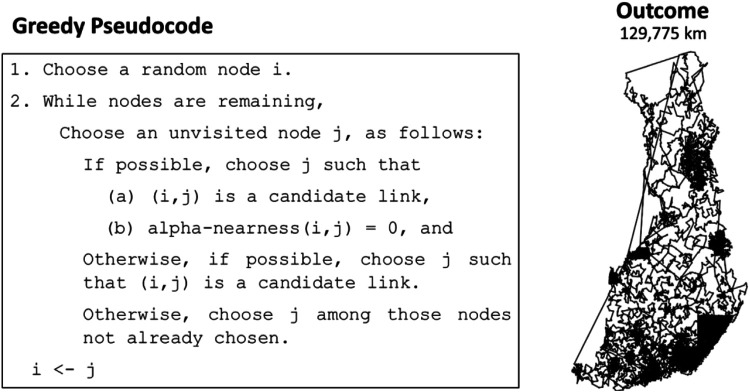
Greedy algorithm used by LKH and the result on the Santa data.

The greedy method used in LKH is explained in Figure 9. Despite the fact that greedy heuristics such as nearest insertion require quadratic time complexity and will not terminate during the course of 1 h on the Santa data, this version of Greedy can be computed efficiently due to the precalculated candidate set, which significantly limits the choices at each step.

As such, LKH was applied to the closed loop variant of TSP and produced a winning tour length of 109,284 km. The other variants are supported with only minor modifications:

A. For the open-loop case, a pseudonode was added. This node was marked as a special node to be recognized by LKH, and all distances from it to all others were considered to be equal to zero.

B. For the fixed-start case, the same pseudonode was added as in (A), but it was forced to be linked to Santa's home.

C. For the *k*-TSP case, multiple pseudonodes were added to the instance and considered to be depots in the multi-TSP variant of LKH.

Analyses of the generated solutions can be found in the *Evaluation* section.

### Tilo Strutz

**FIGURE 10:**
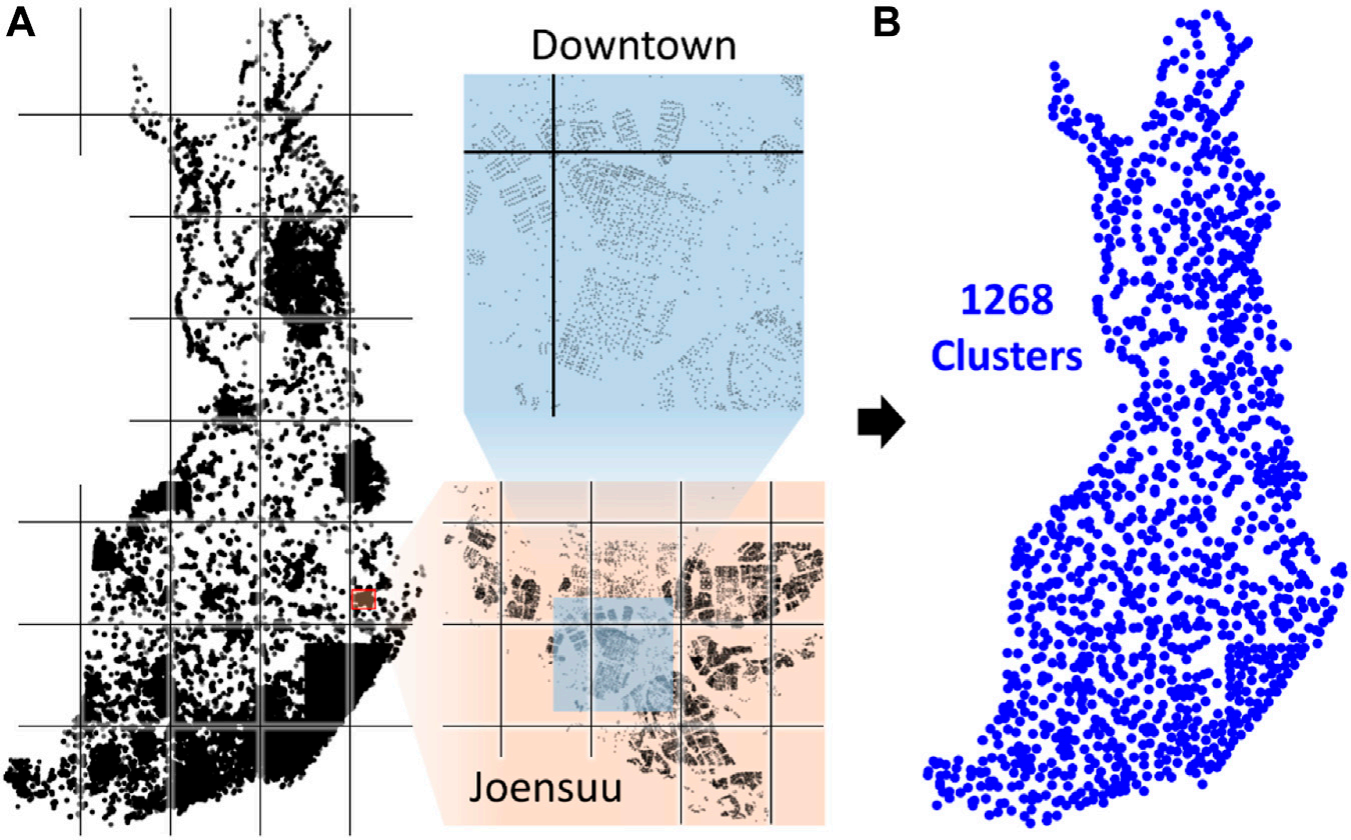
Grid-based division demonstrated **(A)** and the centroids after repartitioning **(B)**.

Tilo Strutz's implementation is called DoLoWire, and its detailed description is documented in his published work (Strutz, 2021). It first clusters the points using a grid-based method. Because the data are not uniformly distributed, the grid adds more cells in regions with higher point density in an attempt to make the size of the subproblems smaller to be solvable in time. To control the number of clusters, DoLoWire has a parameter setting for the maximum value. This parameter was set to 1,500, and a total of 1,268 clusters were generated on the Santa data (see Figure 10). Tilo Strutz noted that this value performed better in practice than at the theoretical best, $\sqrt{N}=1,199$ . Once the cells are generated, a k-means–like step is applied, where centroids are computed based on the data in each cell. Repartitioning is then performed according to the updated centroid locations. This approach models the data better, because it avoids making an overly arbitrary division imposed by the grid (see Figure 10).

**FIGURE 11:**
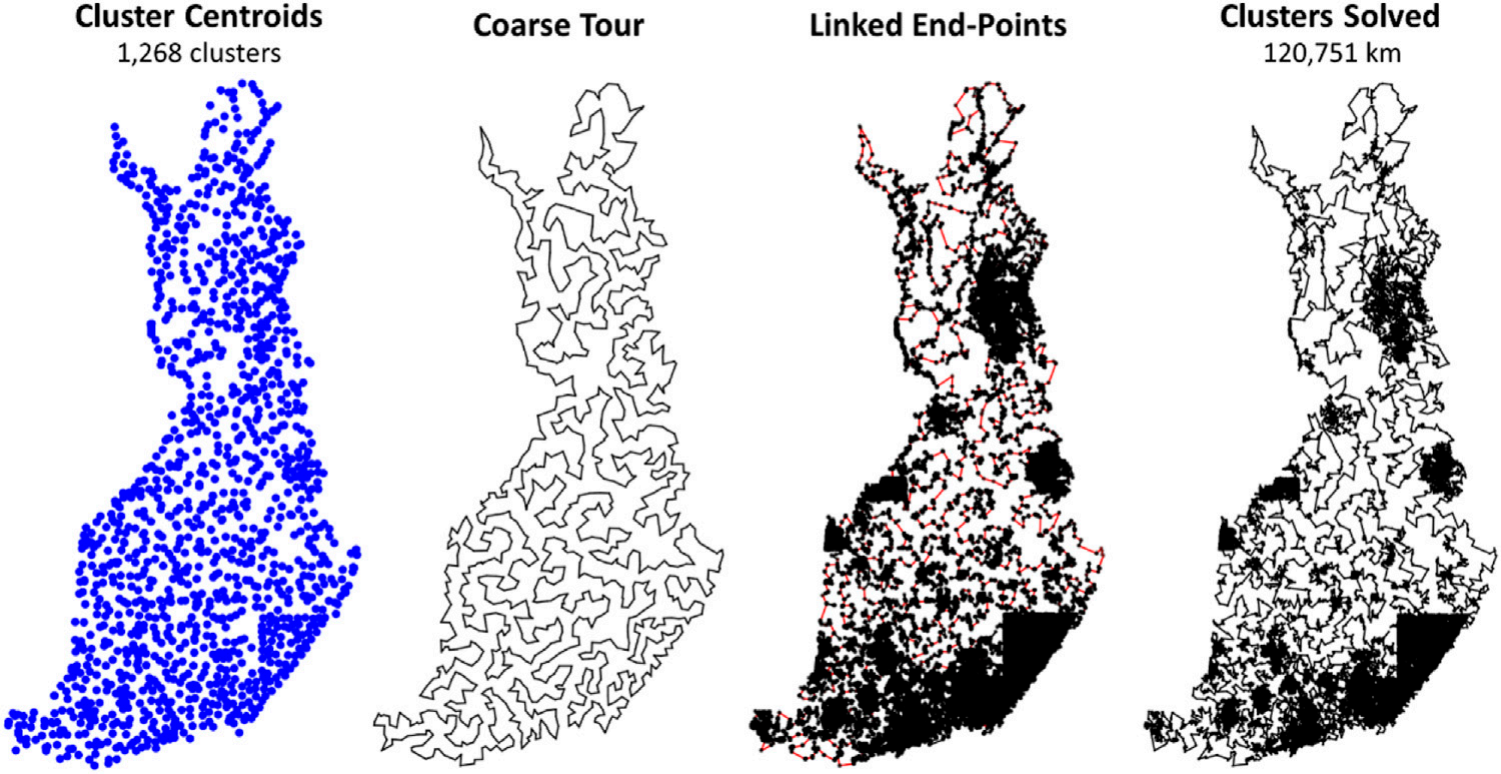
Intermediate steps of DoLoWire visualized.

The cluster centroids are used to generate a coarse tour (Figure 11). This step has a strong impact on the quality of the final solution, because it decides the general order in which the points will be traversed. For this optimization, DoLoWire uses a local search composed of 2- to 3-opt operations with the first improvement strategy: when a better tour is found, it is updated immediately. One important aspect of the algorithm design is that the maximum number of iterations of each cluster is set to 5. This setting was used both when optimizing the coarse tour and at the cluster level. This value controls the processing time of the algorithm in such a way that a lower value decreases the time at the cost of missing some improvements.

With the course tour computed, the next step is to find suitable end points between consecutive cluster pairs. This step is accomplished using a simple search for the nearest pair across the cluster. This search requires quadratic time complexity. However, since the number of clusters is a thousand times smaller than the complete data, this part is not a bottleneck. Figure 11 shows the clusters with the chosen links.

Once the clusters and their connections have been decided, the algorithm optimizes each cluster individually. It uses the same 2- to 3-opt–based local search using the fixed start and end points. The optimization can stop at a prior iteration when 2- and 3-optimality is already achieved. This process takes approximately 40 min (see Figure 11, right).

The remaining 20 min are used for fine-tuning. This step is accomplished by splitting the tour into 500 long nonoverlapping segments and optimizing the tour within each of them. This step is applied in parallel and receives a strong boost here due to our hardware system being composed of 48 cores. This method is the only submission to use parallelization in some way. A final closed loop tour of 115,620 km is reached.

To obtain the other variants, the closed-loop tour was postprocessed as follows:

– Open Loop: Cut the longest link;
– Fixed start: Cut the longest link connected to Santa's home;
– *k*-TSP: Cut the longest *k* links.

Analysis of the generated solutions can be found in the *Evaluation* section.

### UEF

Our method turns out to be very similar to Tilo Strutz's submission, and below, we merely discuss the differences in their design. We use k-means instead of grid-based clustering and find a much larger number of clusters (12,764 > 1,268). The reason is that our TSP solver, which uses random mix local search (Sengupta et al., 2019), was designed for an order of magnitude smaller instances than what would be obtained by dividing the data into √N clusters. Specifically, it can handle at most a few hundred nodes in a reasonable time.

**FIGURE 12:**
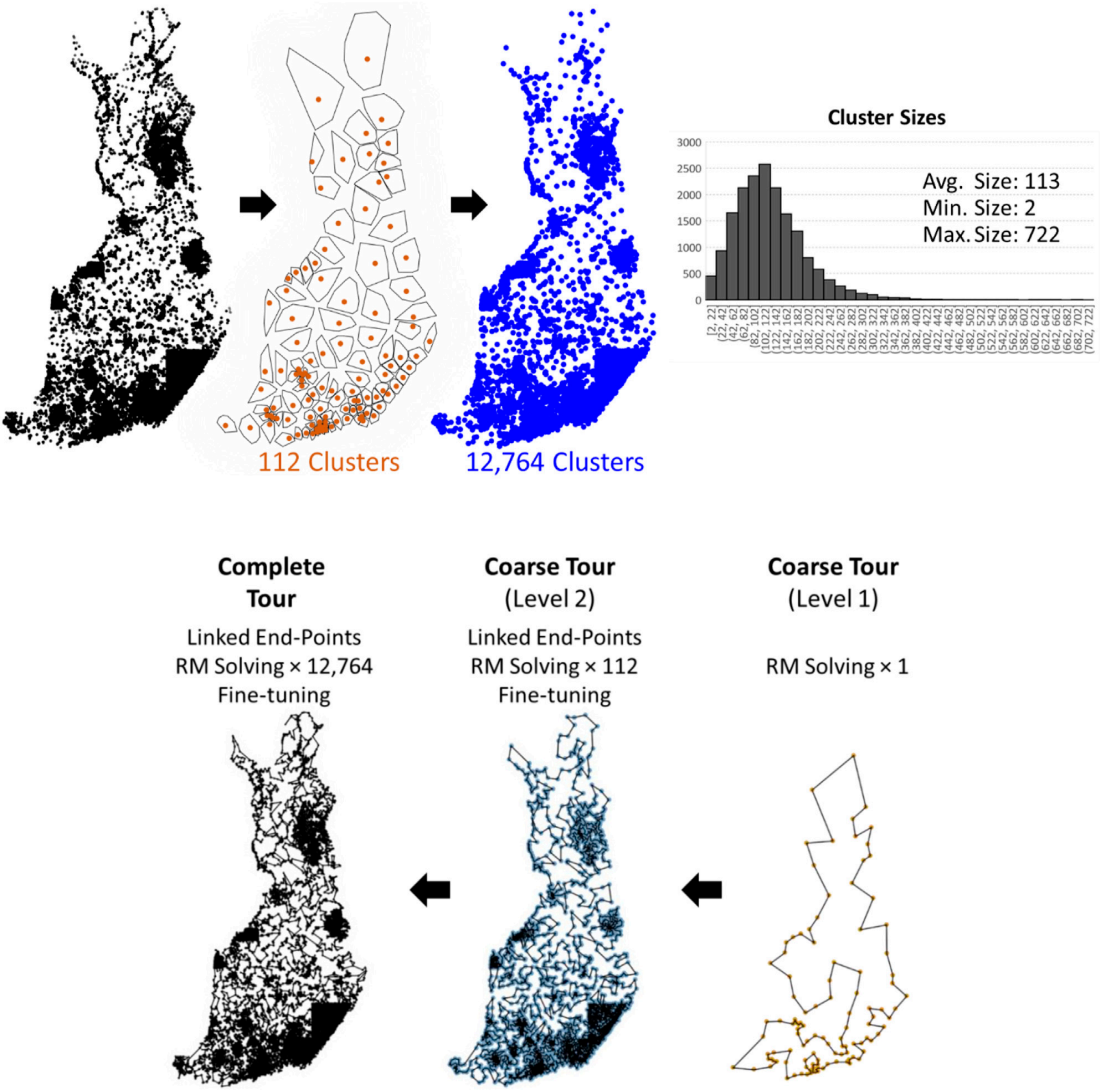
Two-level clusters produced by k-means applied first with *k*_1_ = 112 and then repeated for each cluster with *k*_2_ = 114 on each of the 112 clusters. A distribution of the cluster sizes is also shown. TSPDiv intermediate steps.

K-means takes O(*Nk*) and becomes slower with the increasing number of clusters (*k*). We therefore apply two-level clustering with which we first cluster the data into $k_{1}=\sqrt[{3}]{N}$ = 112 groups, which are then clustered further by a second round of k-means that makes the final number of clusters proportional to $k_{2}=\sqrt[{3}]{N^{2}}\approx \;12,764$ (see Figure 12). The first step is followed by finding the end points and solving the TSP within each of these 112 clusters in Level 2. A fine-tuning step is applied where consecutive clusters are grouped together and optimized jointly. The result is a coarse tour that passes through all 12,764 clusters. This process is repeated to generate a complete tour (see Figure 12).

To complete in 1 h, we use the following parameter settings for the random mix local search: five repeats each with *N* × 3,000 local search iterations at all levels; 1 repeat each with *N* × 3,000 iterations at the fine-tuning stage. The initial tour in the beginning is random. The final tour length produced in this way is 124,162 km before fine-tuning and 122,226 km after fine-tuning.

We obtain the other variants as follows:

– Open Loop: Do not close the coarse tour.
– Fixed start: Force Santa's home and the corresponding clusters to be the first.
– *K*-TSP: Cluster the data into *k* groups by k-means and solve them independently.

**FIGURE 13:**
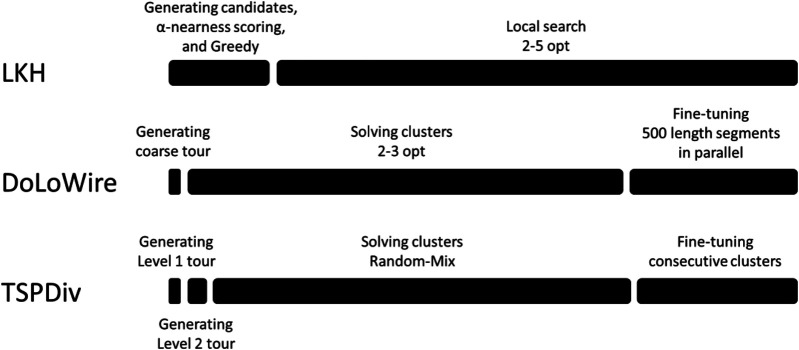
Profiling of submitted methods over the course of 1 h.

We note that in the case of large-scale instances, unlike as reported in Sengupta et al. (2019), the Link swap operator does not play a significant role because it operates with the end points, which have only minor contributions to the very long TSP path. It also does not apply at Level 2 within the clusters because their start and end points are fixed when connecting the clusters. It also does not apply to the closed-loop case. For these reasons, it has been disabled elsewhere except at Level 1 in the open-loop and in the *k*-TSP cases. The components and the processing time profiles of all of the submitted methods are summarized in Figure 13 and Table 1.

**TABLE 1:** Summary of the submitted methods.

	Keld Helsgaun	Tilo Strutz	UEF
Method:	LKH	DoLoWire	TSPDiv
Localization	Neighbors	Grid	Clusters
Local search	Best improvement: 2- to 5-opt with alpha-prioritization	First improvement: 2- to 3-opt	First improvement: 2-opt, node swap and Link swap
Dividing	—	Grid-based clustering	Two-level k-means
Merging	—	Two nearest	Two nearest
Fine-tuning	—	Fixed-length segments (500)	Consecutive cluster pairs
Other variants	Pseudonode	Postprocessing	Pseudonode

## Evaluation

**FIGURE 14:**
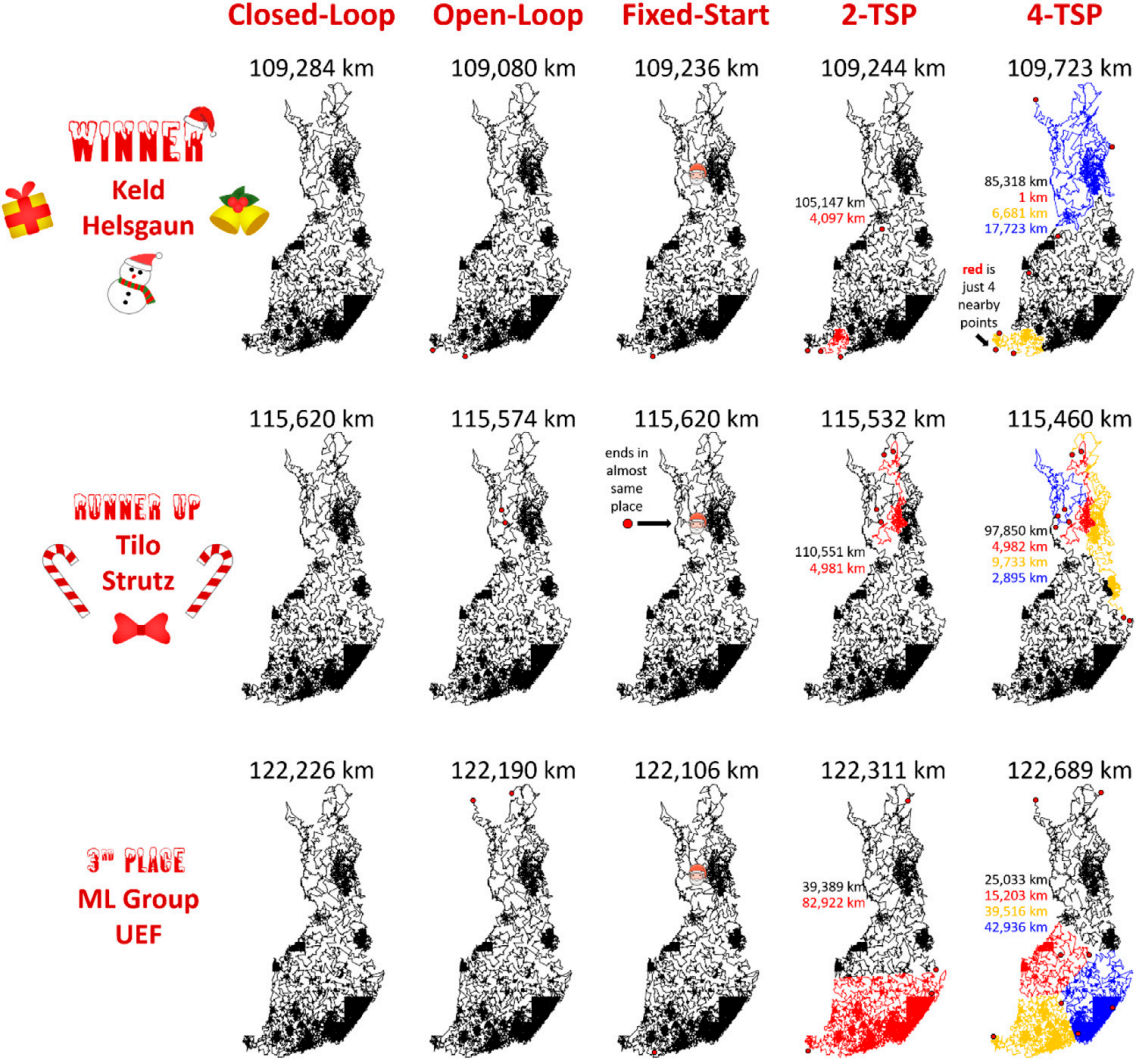
Summary of the results of the Santa competition. The difference between the 1st (109,284 km) and the 3rd (122,226 km) is approximately 12%. When repeated 10 times, the standard deviations of the results were only 98 km (LKH), 47 km (DoLoWire) and 143 km (TSP Div), which shows that the effect of randomness is negligible, which originates from the large number of trial operators applied and the large problem size. According to ANOVA, the results were statistically significant (*p* < 10^30^ in all cases).

**FIGURE 15:**
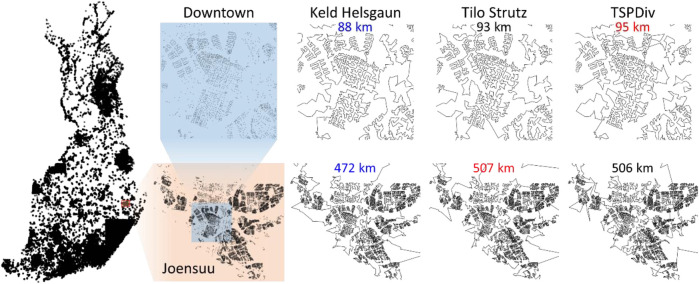
Tours of each of the submitted methods zoomed into view in the city of Joensuu, downtown area (above), and the city area including suburban areas.

The results of the submitted methods are summarized in Figure 14. The main observation is that the performance difference between the methods is clear. The tour length of LKH is 109,284 km, and it is clearly the shortest of all. The gap to the second best (DoLoWire) is approximately 6% and to the third best (UEF) is 12%. What is surprising is that LKH does not use any divide-and-conquer technique to reach the 1-h time limit. A detailed example of the optimized tours is shown in Figure 15.

The results for the open-loop and fixed start point variants have the same ranking. The results of the *k*-TSP case show the importance of a proper task definition. The purpose was to use Santa's helpers to deliver all of the goodies as fast as possible, but the exact objective was not defined. As a result, LKH and DoLoWire minimize merely the total length of all of the subtours, while a more appropriate objective function would have been to minimize the length of the longest subtour. LKH provides four subtours with lengths that vary from 1 to 85,318 km, which is not meaningful for Santa's task. The UEF submission was the only submission that provides a somewhat balanced workload with subtour lengths that vary from 15,203 to 42,936 km.

### Component-Level Analyses (Closed Loop)

#### Localization

LKH was powerful enough to provide the winning tour by solving the problem as a whole without division into subproblems. This finding implies that the use of a neighborhood graph is important. It could miss some links that belong to the optimal tour, but according to the results, its effect is still much less than the effect of the divide-and-merge strategy. It was reported in the work by Peterson (1998) that 75% of the links in an optimal TSP path also appear in the MST and 97% in the XNN graph, which is a subgraph of the much larger Delaunay graph used in LKH. Thus, the chosen neighborhood graph is likely to not miss much.

**FIGURE 16:**
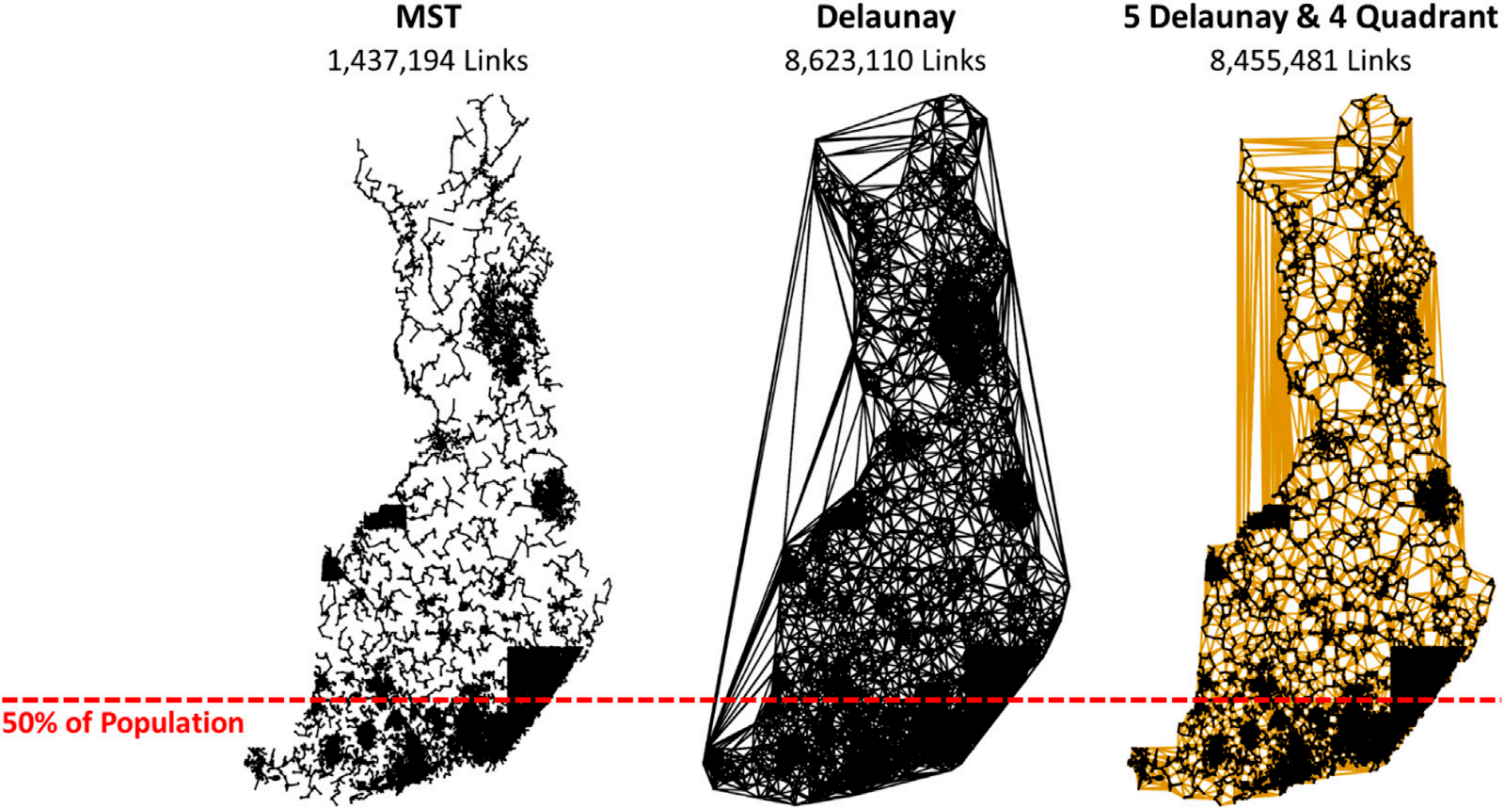
Different graphs and the corresponding number of links. Most people in Finland live in the South, where data are denser than those in the North.

There are also other nearest neighborhood graphs, such as KNN, XNN, ε-epsilon neighborhood, Delaunay, Gabriel, MST, and *k*-MST. Some of them require parameters such as the number of nearest neighbors (KNN), rounds of the algorithm (*k*-MST), or size of the neighborhood (ε-neighborhood). Setting their values larger would increase the number of connections, which would reduce the probability of missing good links but would also increase the search space and slow down the search. This methodology needs to be balanced somehow. Another issue is the connectivity. Other graphs (XNN, Delaunay, Gabriel, and MST) guarantee connectivity and do not require any parameters. Their sizes vary in such a way that MST ⊂ Gabriel ⊂ Delaunay; Delaunay provides the largest number of links (see Figure 16).

Keld Helsgaun considered a hybrid of Delaunay and quadrant links when building the graph. The quadrant graph is formed using nearest neighbors in each of the four quadrants centered at a given point. This method was previously shown to work well in clustered data (Helsgaun, 2000), which is the case with geographical instances such as ours, where cities form clear, dense clusters. Only a maximum of five Delaunay links and four quadrant links per node were considered. The question of how to choose these values for the best performance remains an open problem.

We experimented with LKH with the complete Delaunay graph instead of the hybrid combination while keeping all of the other parameters the same. This approach further improved the tour by 0.3% to 108,996 km with the same processing time. This finding shows that the choice of the neighborhood graph still has room for improvement. Although the data show clear clusters indicating that the hybrid combination of links should work well, other characteristics also have an impact. Finland shows significant differences in density from south to north. Approximately 20% of the population lives in the Helsinki metropolitan area and 50% in south Finland^8^ (see Figure 16). Finland also has thousands of lakes that have a significant impact on optimal routing, especially in East Finland.

Clustering allows solving larger-scale instances more efficiently, but it also has a role in localizing the search space by limiting the search within the cluster. Another benefit is that it supports direct parallelization, where one cluster could be solved by one processor if enough CPU resources are available.

#### Local Search

We compare the three search strategies:

– 2- to 5-opt with neighbor candidates and alpha prioritization (Keld Helsgaun)
– 2- to 3-opt (Tilo Strutz)
– 2-opt, Relocate, and Link swap (UEF)

**FIGURE 17:**
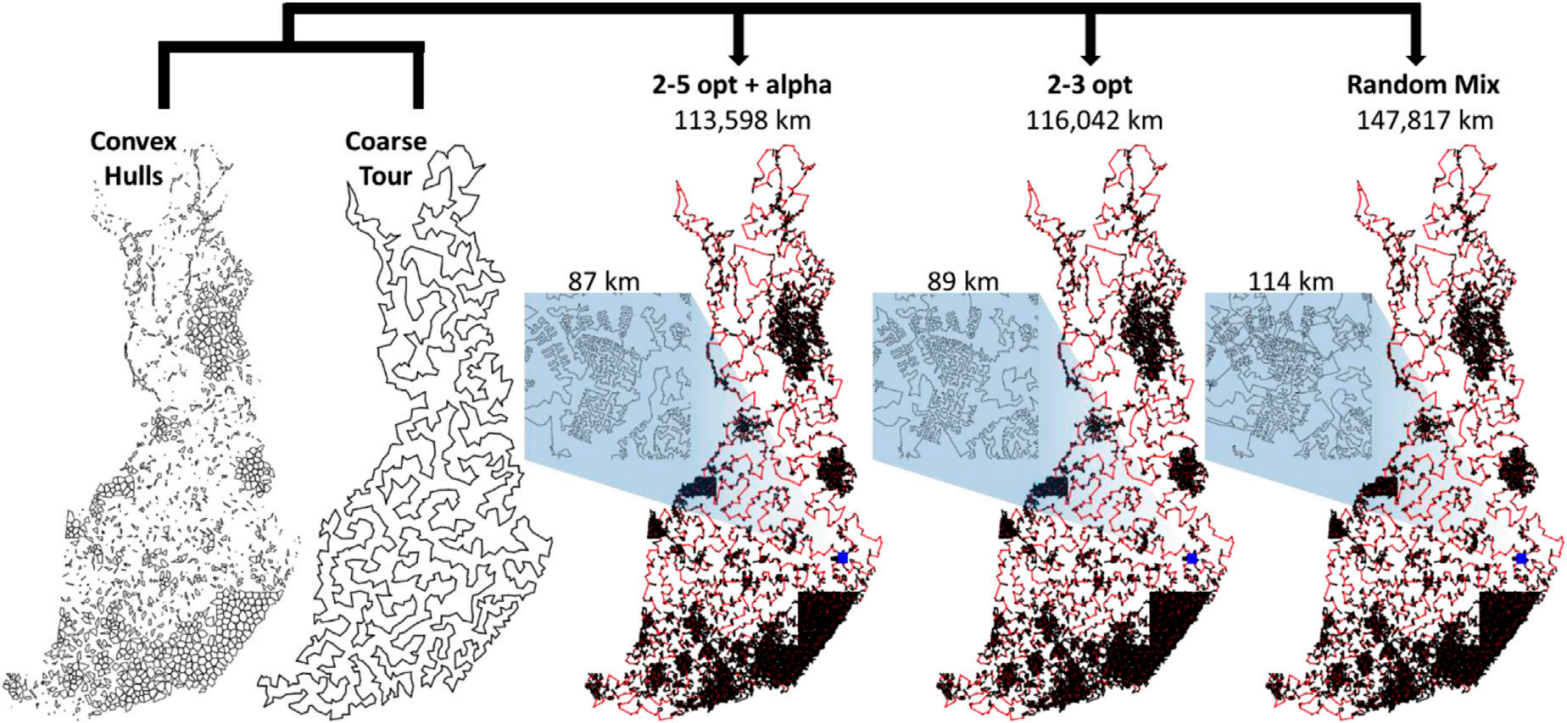
Results when applying 3 different local search strategies when optimizing the same set of clusters and merging along the same coarse tour. The between-cluster connections are shown in red.

We fixed the division method and the grid-based clustering of Tilo Strutz. We also fixed the order of the coarse tour, as shown in Figure 17, in such a way that the resulting solutions would differ only inside the clusters. We attempted to solve the same clusters using all three methods shown above over the course of 40 min to allow for 20 min of fine-tuning (see the *Clustering* section). To complete the computations in approximately 40 min, we set LKH to terminate after at the most 3 s per cluster and set the number of random mix repeats to 1, and the iterations equal to 11,000 × the size of the cluster. For DoLoWire, we used the default settings. All of these settings terminated in approximately 40 min.

From the results in Figure 17, we note that the solution length varies significantly in the three cases. Even though they do not look much different in the zoomed out view, zooming in reveals the limitations of random mix in solving such large cluster sizes properly. Many suboptimal choices are visible. It is good on small instances but never converges with larger instances because of a search space that is too large. The 2- to 3-opt provides better results, and substantially fewer artifacts are visible. The 2- to 5-opt with alpha-prioritization produces the best result despite the extra time required to compute the Delaunay graph for each cluster. The more powerful operator and the selection strategy compensate for the extra time. The result (113,598 km) is better than any of the submitted divide-and-merge variants.

#### Clustering

**FIGURE 18:**
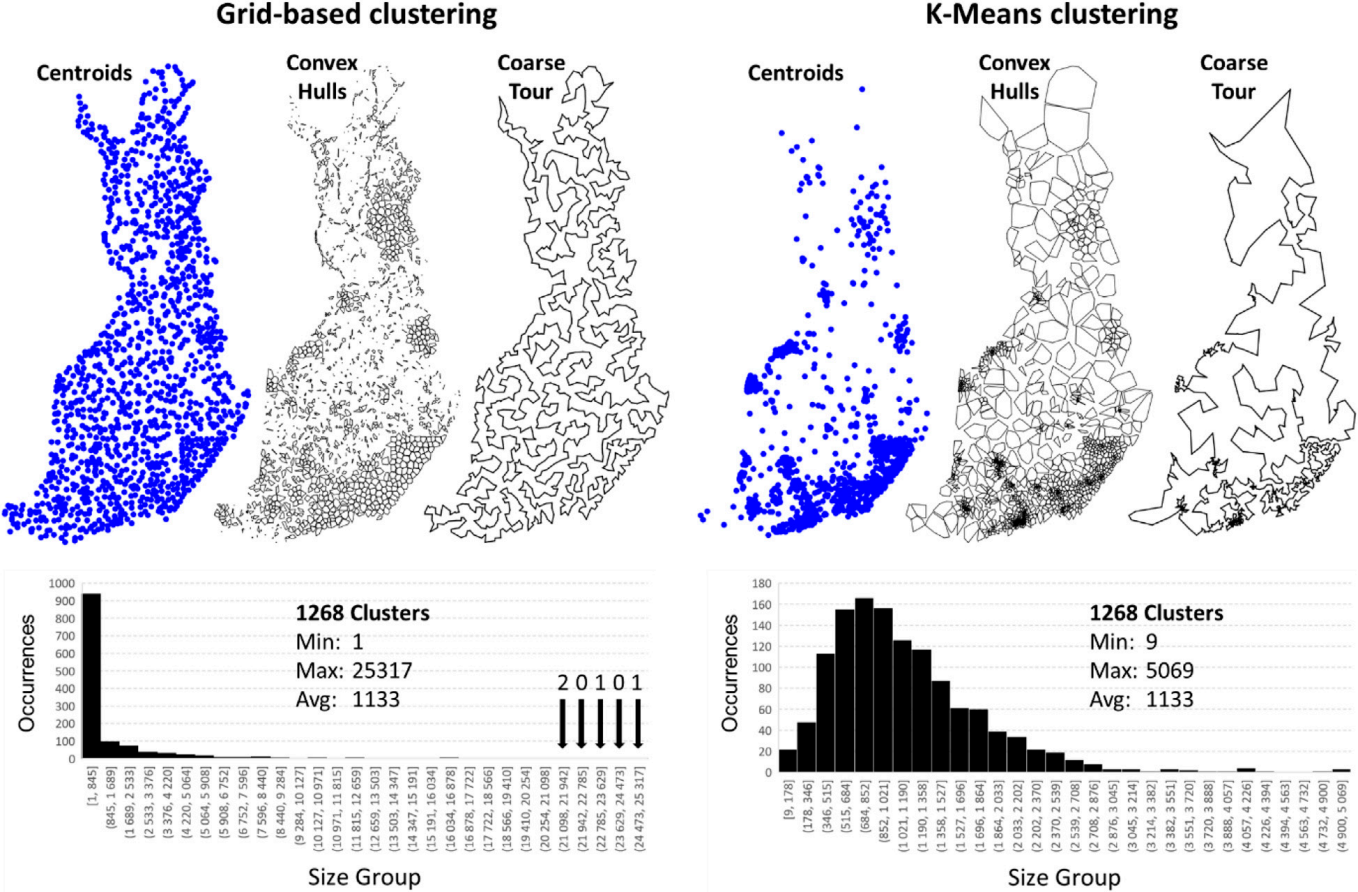
The data clustered by Strutz’ grid-based method **(A)** and k-means **(B)**. The same number of clusters was used in both (*k* = 1,268). Example tours through the centroids are shown. Histograms of the cluster sizes are also shown.

We next compare the two clustering methods (grid and k-means) in Figure 18. Strutz's grid-based method with his parameter choices produced 1,268 clusters, and thus, we also set the same value for k-means to have a fair comparison.

The centroids in the grid clustering are more uniformly distributed over the country, and north Finland is better covered by the clusters than using k-means. This finding occurs because grid-based clustering has a fixed size of the cells independent of the volume. K-means clusters vary more: larger clusters appear in the north, and smaller clusters appear in the south. The coarse tour for the grid clusters has links of roughly equal sizes (distances between the cells) compared to the tour for k-means.

We used LKH to solve the individual clusters. In preparation for the merging step to follow, we fixed the start and end points for each cluster to be the nearest points between the consecutive clusters in the coarse tour. We can impose the fixed start and end points to be used by LKH using the pseudonode strategy and two fixed links.

**FIGURE 19:**
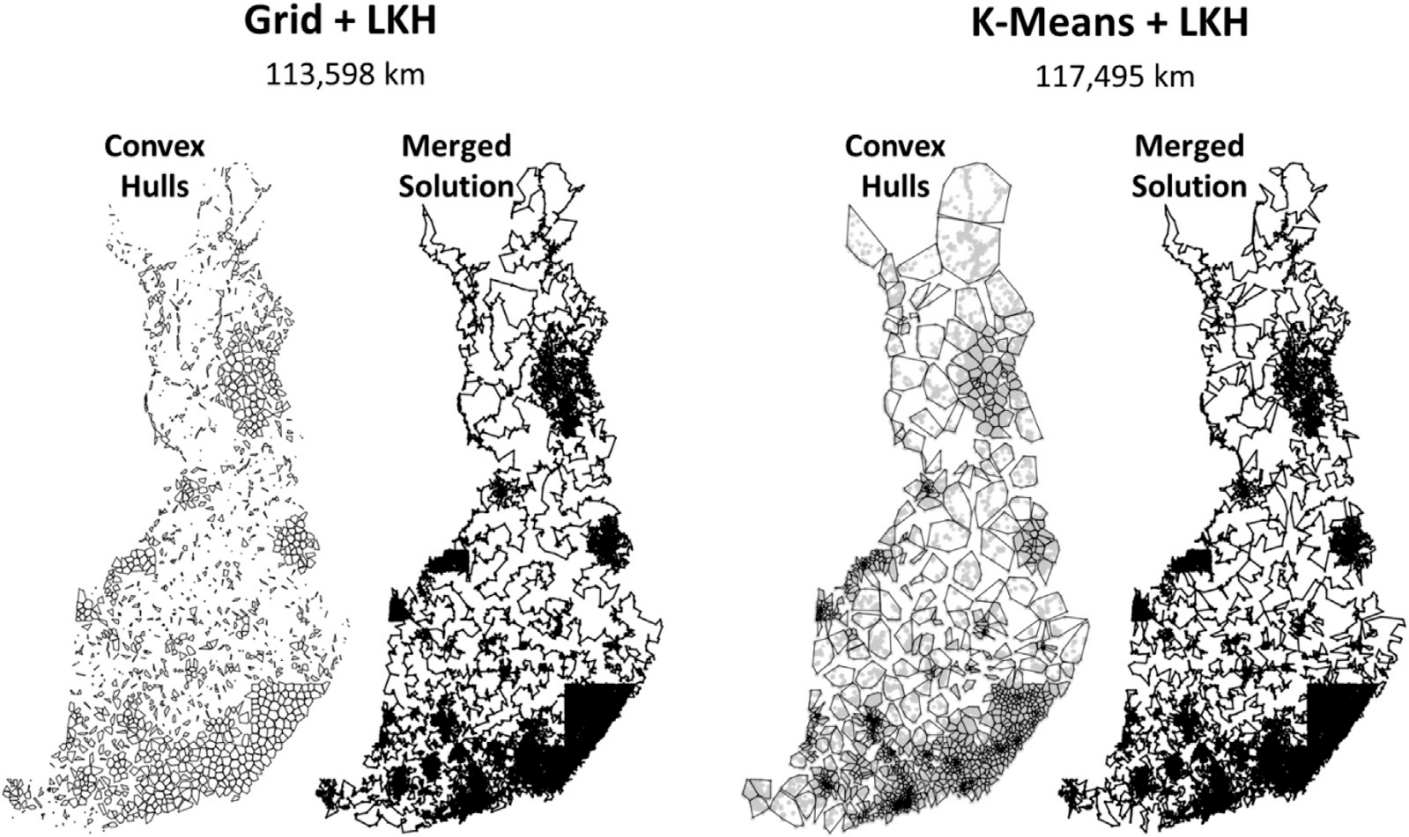
Solutions after solving individual clusters and merging the subtours. The convex hulls of the clusters are given for reference. On the right, data points are also shown to highlight the patterns.

**FIGURE 20:**
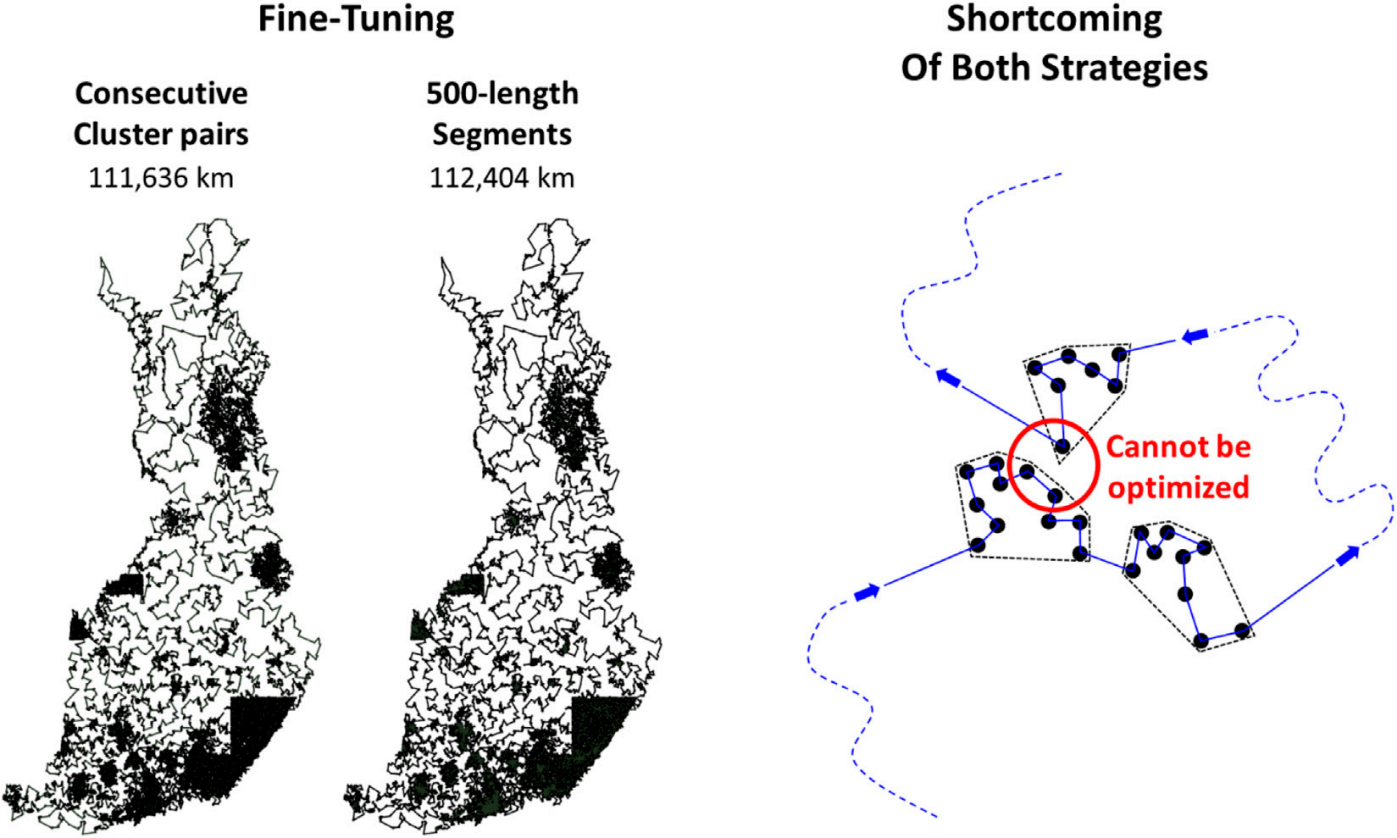
Result of fine-tuning using two techniques and an example where optimization is not possible using either of the two techniques.

The grid clustering produces a better result. The main reason is that it better represents the northern part of Finland. K-means generates clusters with large volumes but loses information about how those points are distributed (see Figure 19). For example, we note that the north-most convex hull contains a visible looping path formed by buildings along main roads; this information is lost when a single centroid is used to represent the cluster. The coarse tour is forced to move west from here, because going back to the south is not possible (returning to the same cluster). The grid-based clusters are smaller here and allow the coarse tour to return to the south. The grid cells are small enough to better preserve these patterns. The merged solution in k-means also has visible artifacts where the solution could be easily improved (see Figure 20).

The south is modeled better by k-means, but the improvement is less significant. The reason is that in high-density areas, there are plenty of alternative choices, and it is easier to find many almost equally good alternatives. In the low-density areas in the north, even a single mistake can have a serious impact on the overall tour length, because there are fewer alternatives and unnecessary detours can mean tours that are hundreds of kilometers longer. In other words, sparsely populated areas are more critical for performance than high-density areas.

We also experimented by changing the parameters that control the cell size, but the value of *k* = 1,268 appears to be locally optimal, and it is difficult to find significant improvement. There are simply too many possible parameter combinations to test, and the high computing time is a serious restriction to running large-scale testing. The selected value is quite close to the theoretical best $\sqrt{N}=1,199$ , and thus, we do not expect significant improvement here.

In conclusion, grid clustering is better for this purpose because it better models the low-density areas where mistakes have a more severe impact on the overall tour length. In the following tests, we fix LKH to use alpha prioritization on a full Delaunay graph to avoid the need for parameter tuning and because it was shown to produce better results than the original heuristic used by LKH (5 Delaunay, 4 Quad hybrid).

#### Fine Tuning

We tested the two fine-tuning strategies by applying the LKH solver for subsets produced as follows:

– Nonoverlapping segments of length 500.
– Adjacent cluster pairs.

We applied fine-tuning for the Grid+LKH combination shown in Figure 19 (left). We set a maximum processing time of 2 s per subset. Each time, the initial tour was simply the combined subtours of the two segments (or clusters). Both fine-tuning strategies terminated in approximately 20 min each. The results are shown in Figure 20. The adjacent clusters strategy is better. However, both strategies optimize merely among the pairs of points that are near to each other in the tour sequence but not necessarily in the space. We expect a better fine-tuner to be constructed by selecting the subsets from sequences (or clusters) nearby in the space but far away in the tour sequence.

#### Summary of the Results

The main results are summarized in Table 2. The first observation is that localization is more effective than dividing. LKH (109,284 km) achieves the best results even if fine-tuning was applied after clustering (111,636 km). The role of clustering therefore remains as facilitating parallel processing. The second observation is that the localization can be improved using Delaunay alone instead of combining it with the quadrant neighbors as in the submission. The third observation is that the effect of fine-tuning is most significant with DoLoWire (120,751 vs. 115,620 km) because of the effect of parallelization. Without parallelization, UEF fine-tuning method (adjacent clusters) is better. Among the other parameters, the number of clusters is also remarkable. Too many clusters are bad with LKH (121,020 vs. 117,496 km), whereas too few clusters are bad for Rand mix (122,226 vs. 147,817 km) because the clusters are large, and the local operators lose their effectiveness.

**TABLE 2:** Summary of the results. The baselines of the three submitted methods are in boldface.

	Operator	Localization	Divide	Clusters	Fine-tuning	Result (km)
Greedy	—	—	—	—	—	129,775
**UEF and DoLoWire:**						
UEF (no tuning)	Rand mix	Clusters	K-means	12,764	—	124,162
**UEF**	Rand mix	Clusters	K-means	12,764	Two clusters	**122,226**
UEF (grid clusters)	Rand mix	Clusters	Grid	1,268	Two clusters	147,817
DoLoWire (no tuning)	2- to 3-opt	Grid	Grid	1,268	—	120,751
**DoLoWire**	2- to 3-opt	Grid	Grid	1,268	500 points	**115,620**
**LKH with clustering:**						
LKH (k-means)	2- to 5-opt	Neighbors	K-means	12,764	—	121,020
LKH (k-means)	2- to 5-opt	Neighbors	K-means	1,268	—	117,496
LKH (grid)	2- to 5-opt	Neighbors	Grid	1,268	—	113,598
LKH (grid + tuning)	2- to 5-opt	Neighbors	Grid	1,268	500 points	112,404
LKH (grid + tuning)	2- to 5-opt	Neighbors	Grid	1,268	Two clusters	111,636
**LKH without clustering:**						
**LKH**	2- to 5-opt	Neighbors	—	—	—	**109,284**
LKH (Delaunay)	2- to 5-opt	Delaunay	—	—	—	108,996

## Conclusion

We studied three solutions for a large-scale TSP problem in the Santa Claus challenge in 2020. From the results, we learned a few important lessons. First, large-scale instances have immediate consequences that must be taken into account when designing algorithms for big data. Size of 1.4 M is already so large that even a simple greedy algorithm would take about 3 days with our current hardware to compute because of quadratic time complexity. To solve problems of this size in 1 h, a linear (or close to linear) algorithm is required.

We also draw the following conclusions:

• Spatial localization of the local search operator is most important.
• Local search with *k*-opt is still the state of the art.
• The *k*-opt needs to be extended to 4-opt and 5-opt.
• Current divide-and-merge strategies requires further improvements.
• Parallelization would be an easy addition to speed-up the methods further.

In specific, without the neighborhood graph, the *k*-opt and random mix operators produced three orders of magnitude worse solutions because of the huge search space. Random initialization was another limitation, but thanks to the localization by neighborhood graph, greedy initialization could be calculated efficiently.

While there were only three valid submissions, they were all based on local search. Literature review did not reveal any other method than local search capable of scaling up to data of the size of 1 M. In one article, GA was found to be more effective than local search but only up to *N* = 5,000, and when needed to find the exact optimum (Paul et al., 2019). Probably the strongest evidence of local search being the state of the art is that the method by Keld Helsgaun has held the record for the other large-scale benchmark data set (WorldTSP), almost without a break since 2003.

About the chosen data from OSM: it was fit for the purpose but suffered some artifacts because the coverage of the data varied a lot. While the data is valid, a more accurate building distribution in Finland would be available.^9^

We derived the best divide-and-conquer approach from the components of the submitted variants and reached a solution with a 2% gap to the best method (LKH). This finding is significantly better than the gap values of the two other submitted methods (DoLoWire = 6%, UEF = 12%). The potential of the divide-and-conquer approach comes from the fact that the calculations could be easily performed in parallel using a multiprocessor system. This finding also applies to the fine-tuning step of the 1,267 merged cluster pairs. With a state-of-the-art cloud infrastructure, we could run one task per machine (Mariescu-Istodor et al., 2021), which would bring the processing time from 1 h down to 2 min.

## Data Availability

All data is available in the Santa Claus TSP challenge web page: https://cs.uef.fi/sipu/santa/.

## Footnotes

1
https://www.statista.com/statistics/878354/fedex-express-total-average-daily-packages

2
https://gizmodo.com/can-santa-claus-exist-a-scientific-debate-1669957032

3
https://www.openstreetmap.org

4
http://cs.uef.fi/sipu/santa/data.html

5
http://www.math.uwaterloo.ca/tsp/world

6
https://www.geonames.org

7
http://akira.ruc.dk/∼keld/research/LKH

8
https://www.verkkouutiset.fi/karttakuva-nayttaa-puolet-suomalaisista-asuu-taman-viivan-alapuolella

9
https://www.avoindata.fi/data/fi/dataset/rakennusten-osoitetiedot-koko-suomi
