# Supplementary material for: Solving the Large-Scale TSP Problem in 1 h: Santa Claus Challenge 2020
Source: Front Robot AI. 2021 Oct 4;8:689908. doi: 10.3389/frobt.2021.689908 (PMC8520904; doi:10.3389/frobt.2021.689908)

## Slide 1
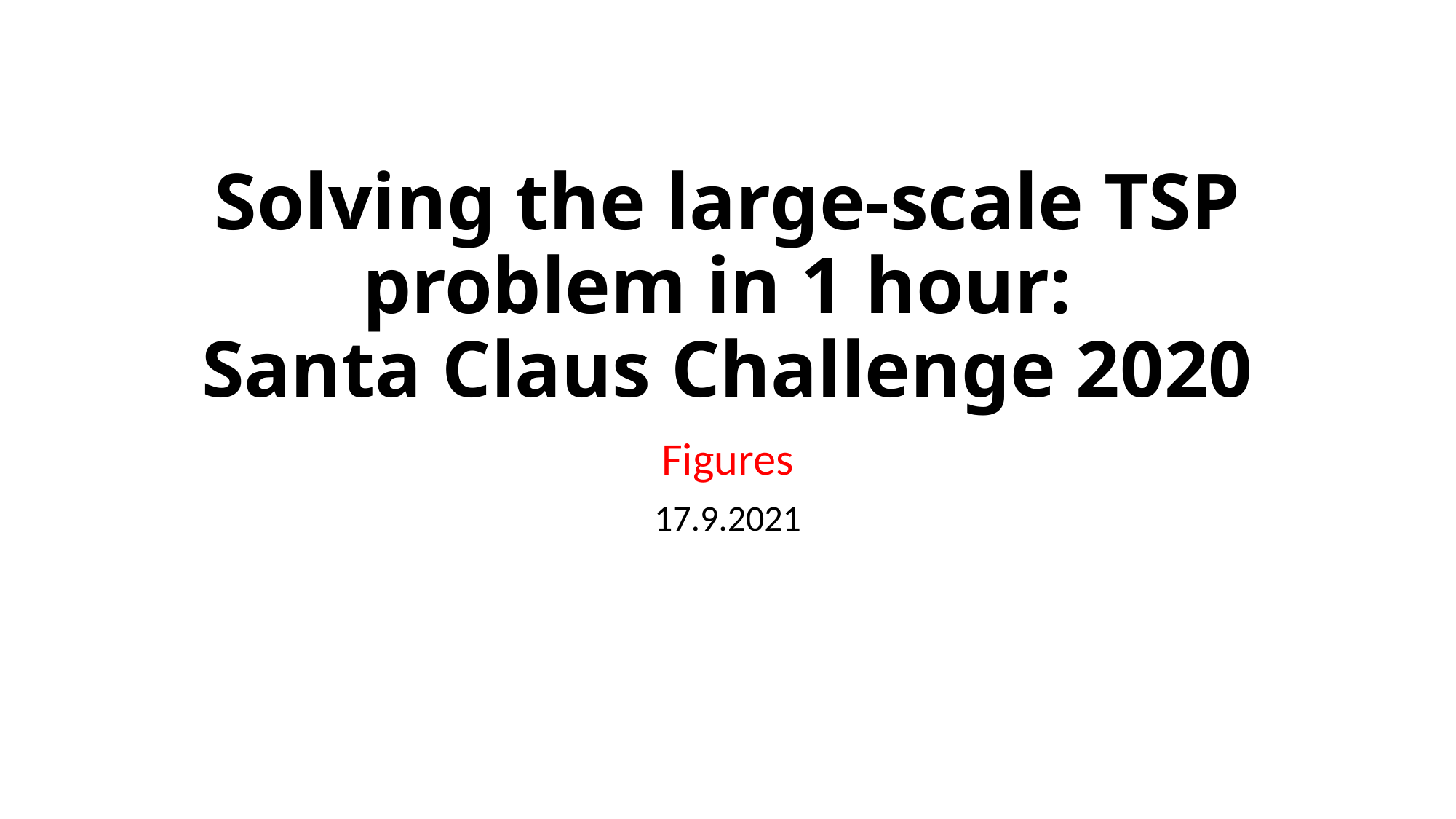

# Solving the large-scale TSP problem in 1 hour: Santa Claus Challenge 2020
Figures
17.9.2021

## Slide 2
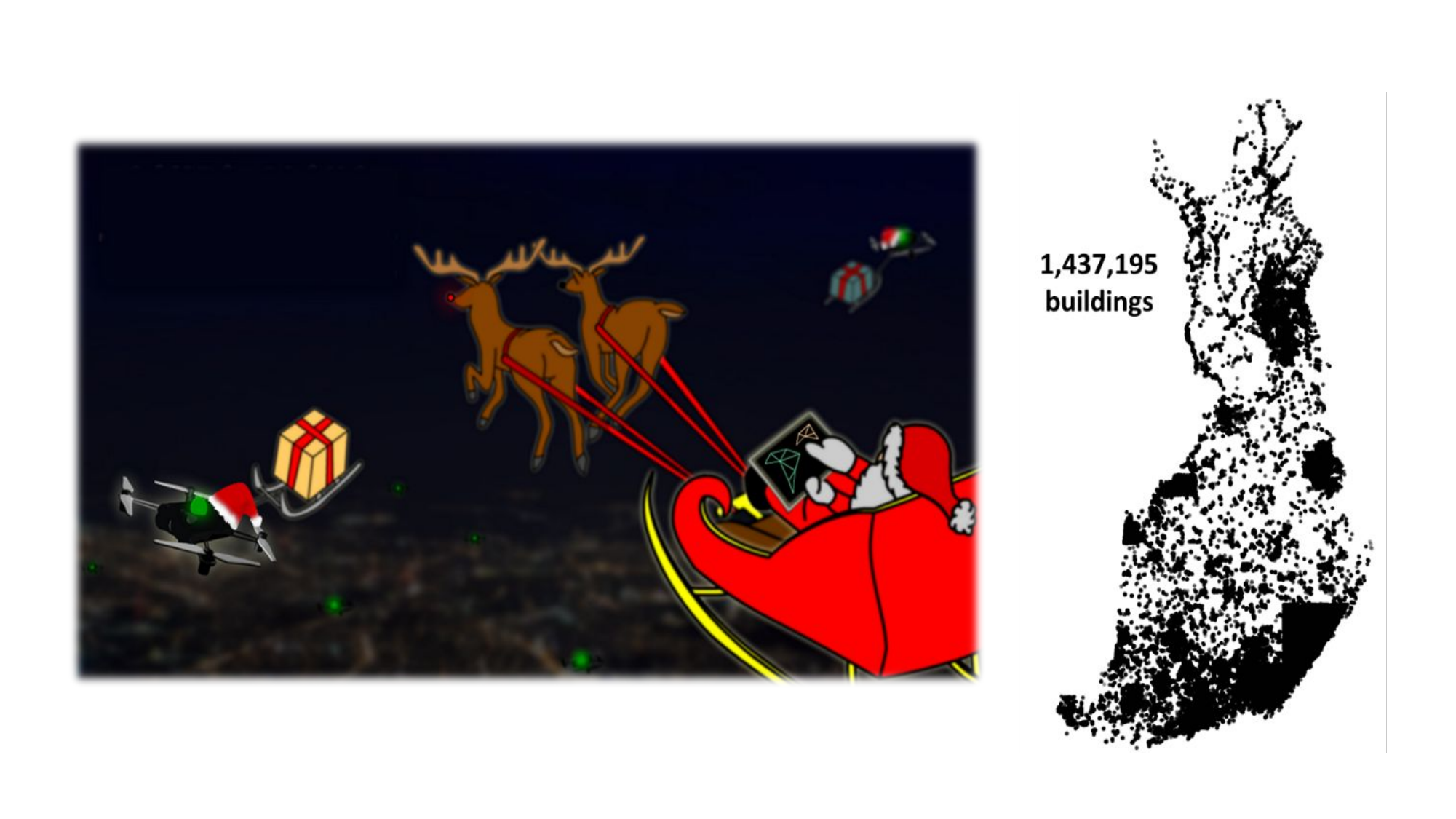

## Slide 3
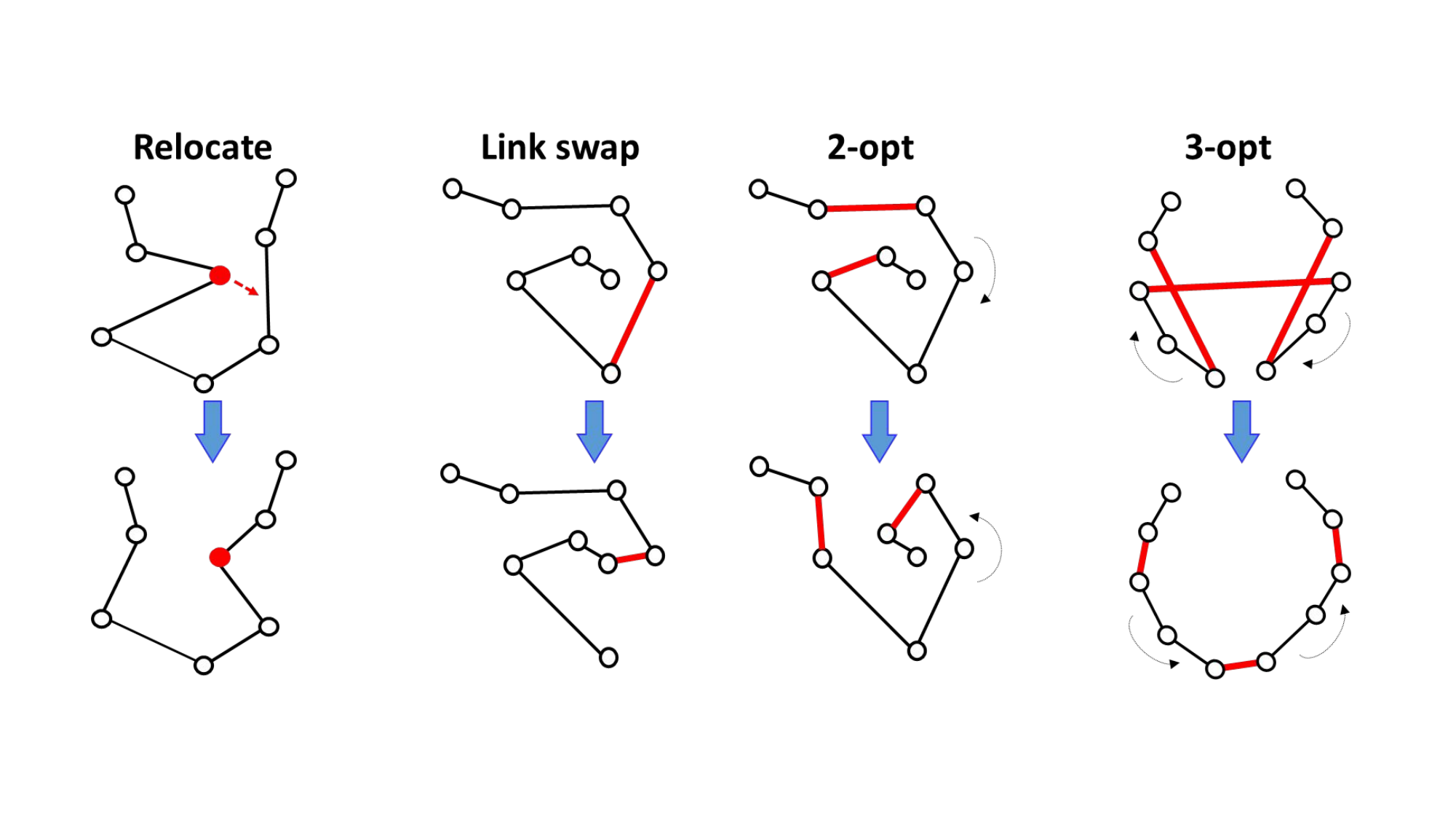

## Slide 4
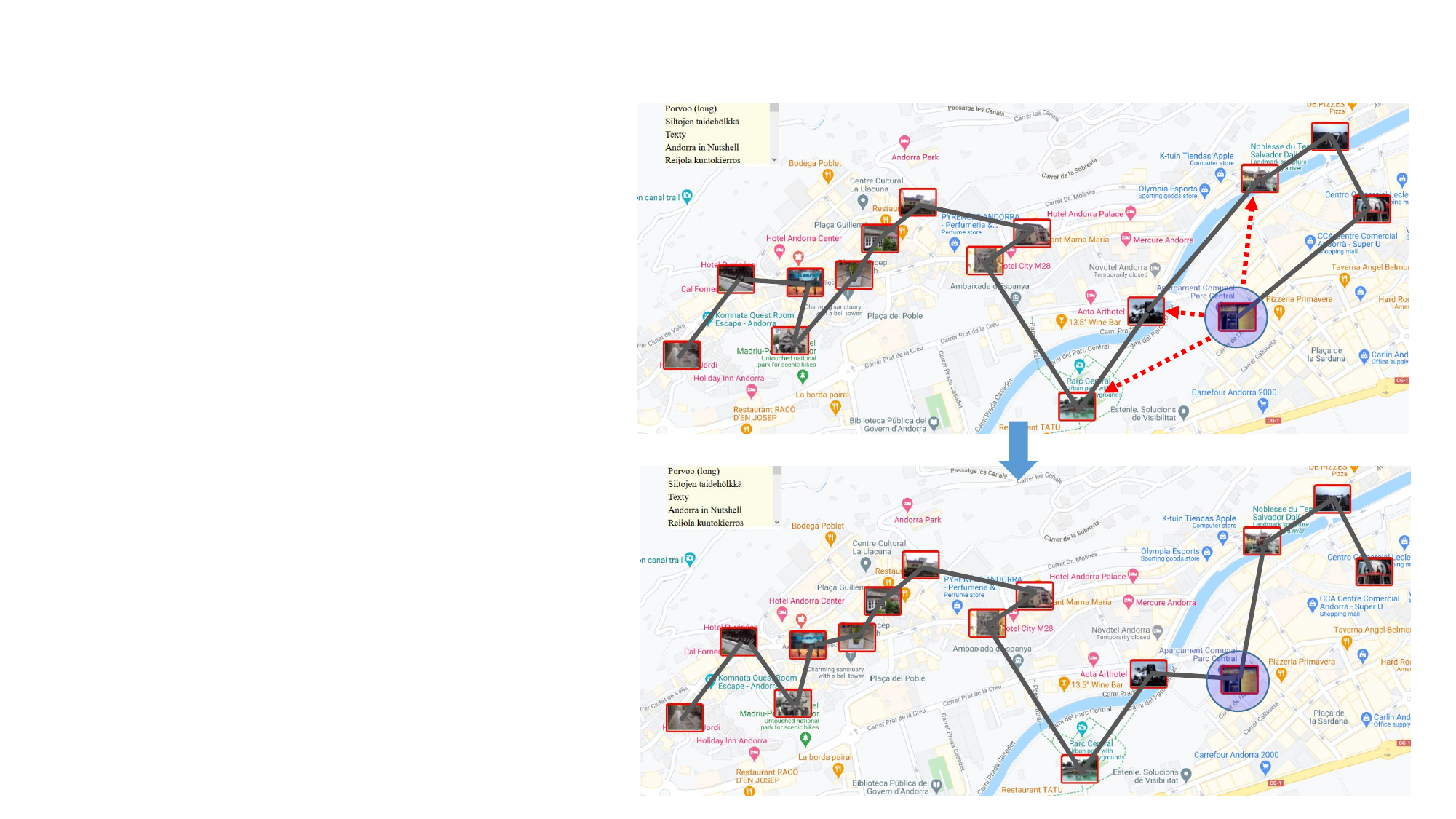

## Slide 5
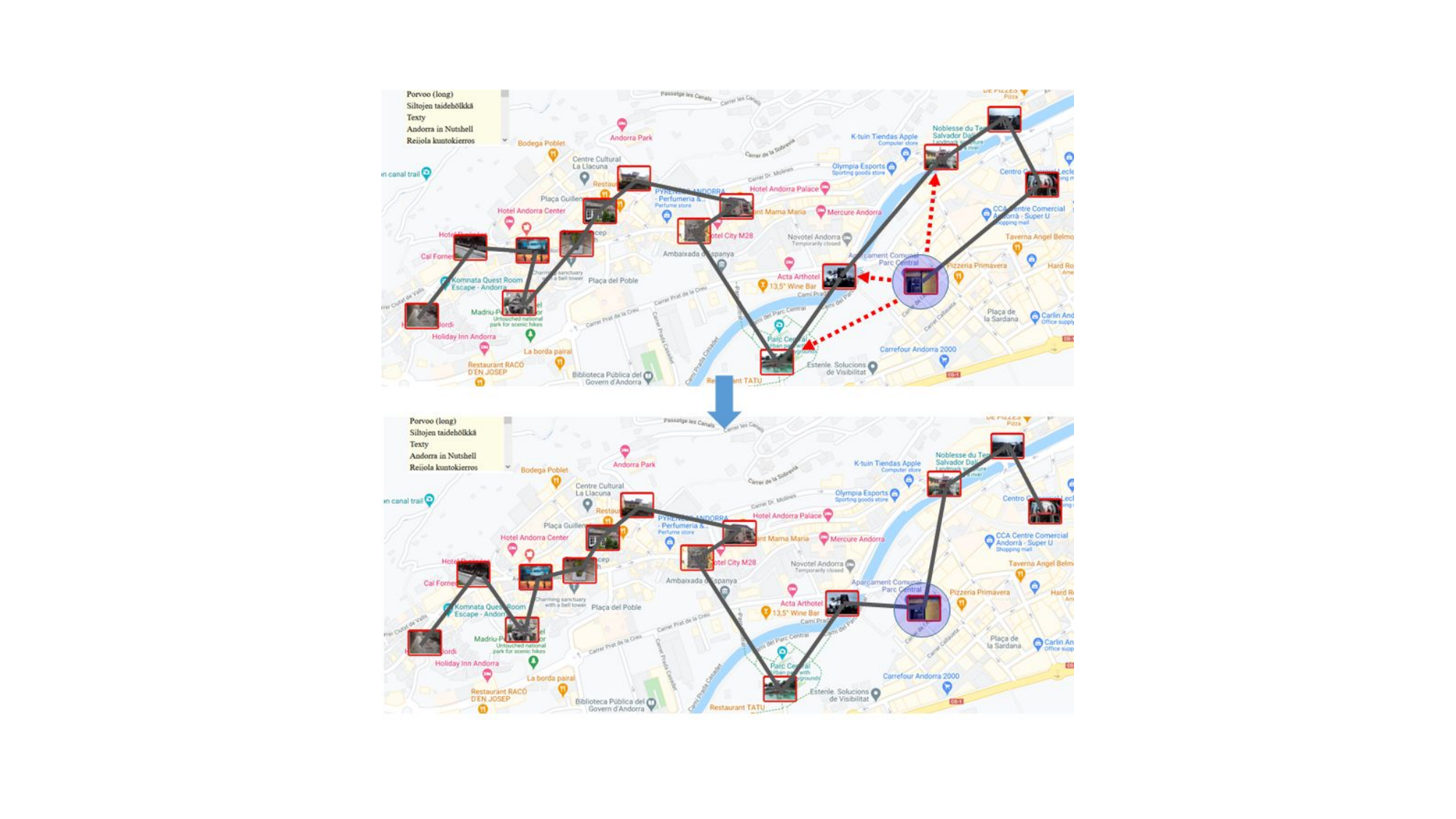

## Slide 6
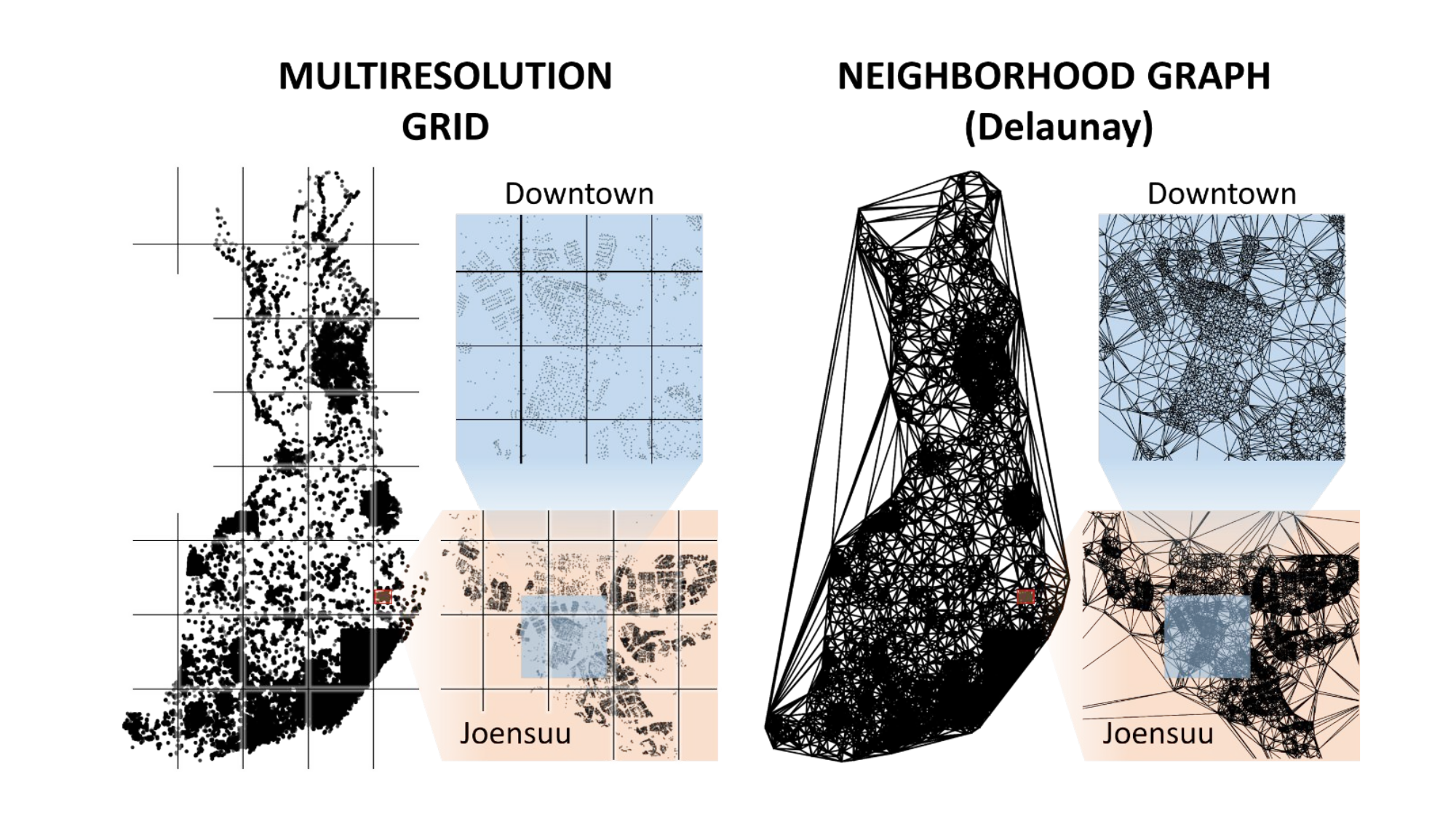

## Slide 7
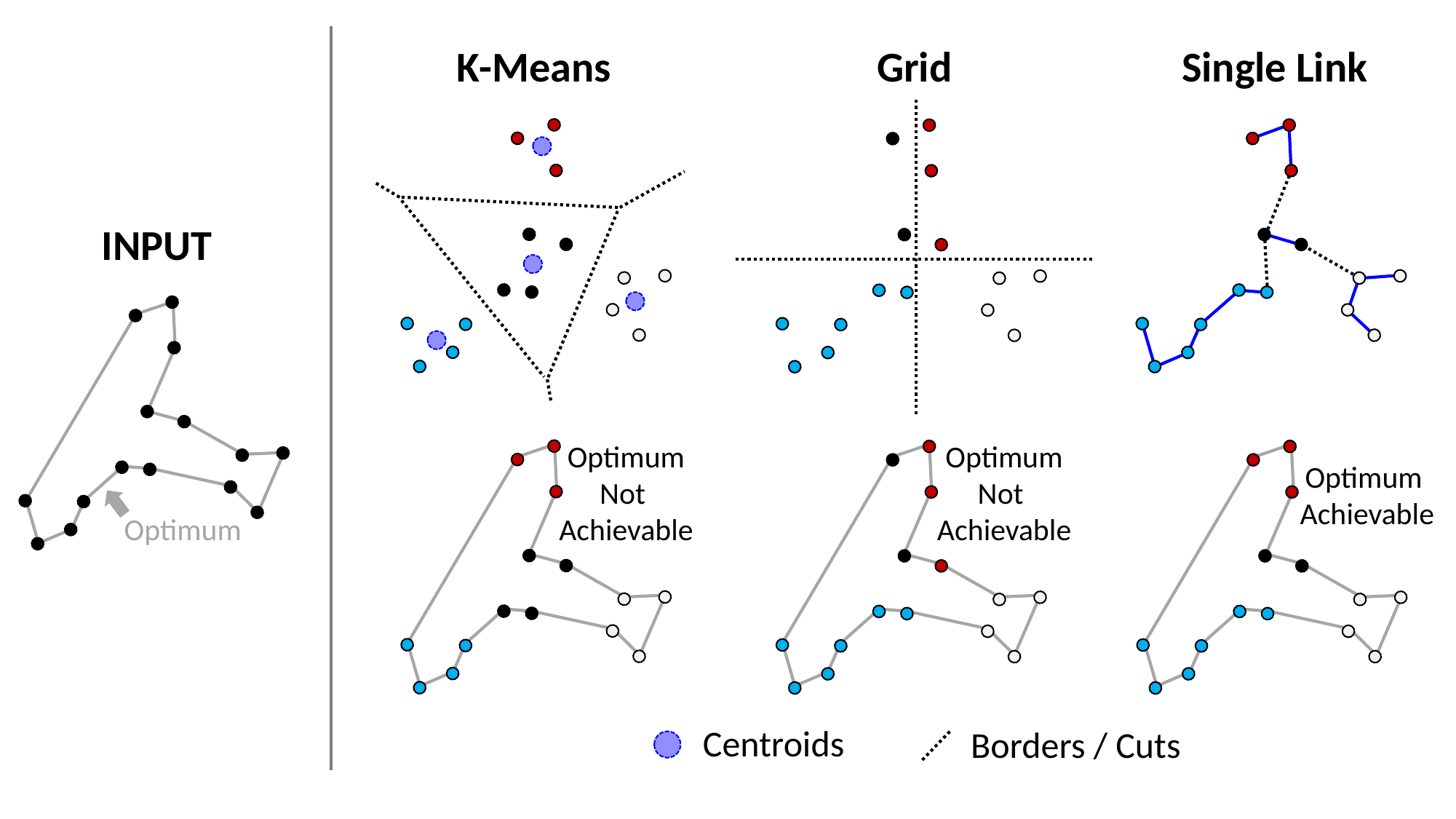

K-Means
Grid
Single Link
INPUT
Optimum
Not
Achievable
Optimum
Not
Achievable
Optimum
Achievable
Optimum
Centroids
Borders / Cuts

## Slide 8
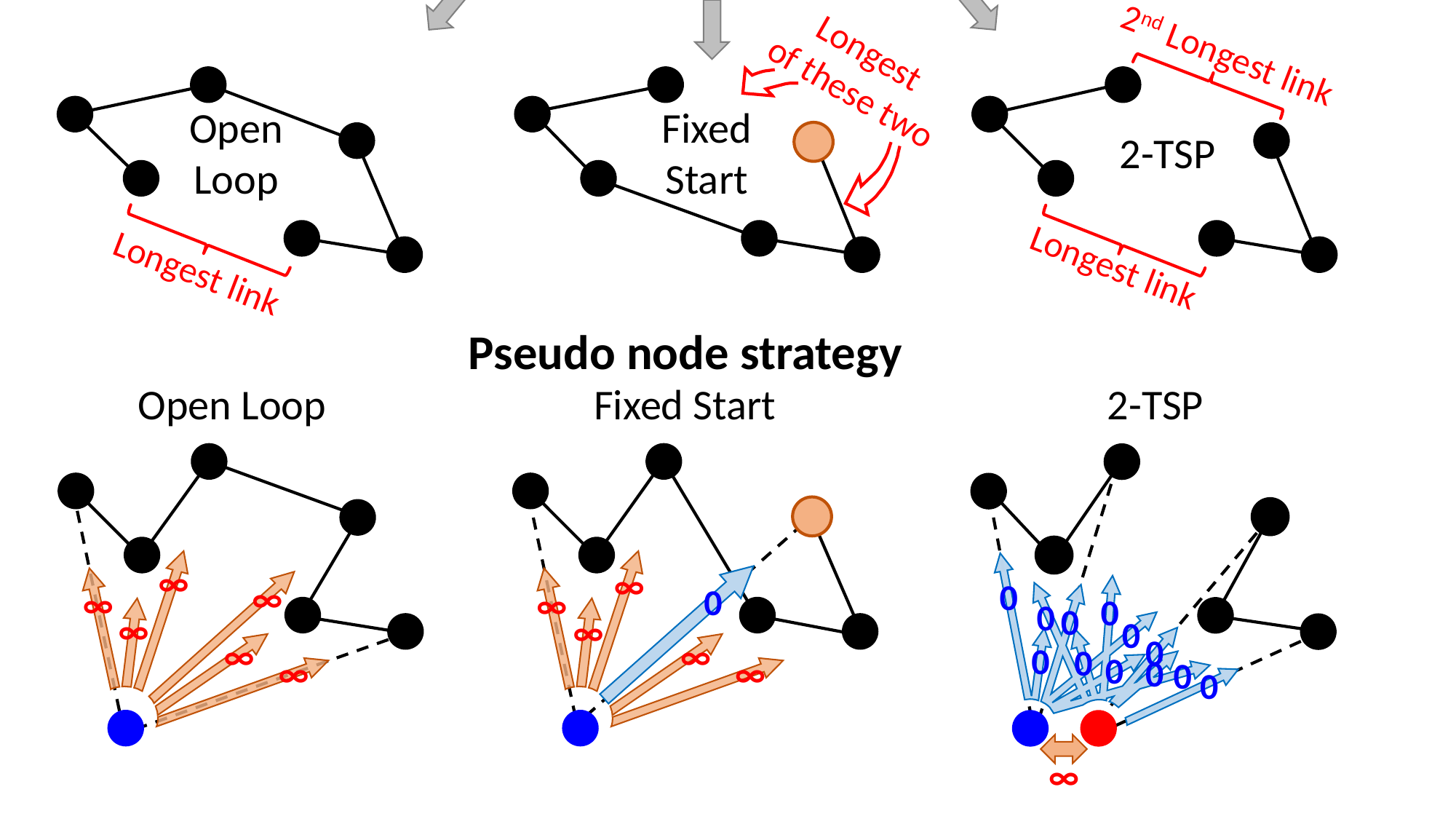

Cutting strategy
Closed
Loop
Longest
of these two
2nd Longest link
Open
Loop
Fixed
Start
2-TSP
Longest link
Longest link
Pseudo node strategy
Open Loop
Fixed Start
2-TSP
∞
∞
∞
∞
∞
∞
∞
0
∞
∞
∞
∞
0
0
0
0
0
0
0
0
0
0
0
0
∞

## Slide 9
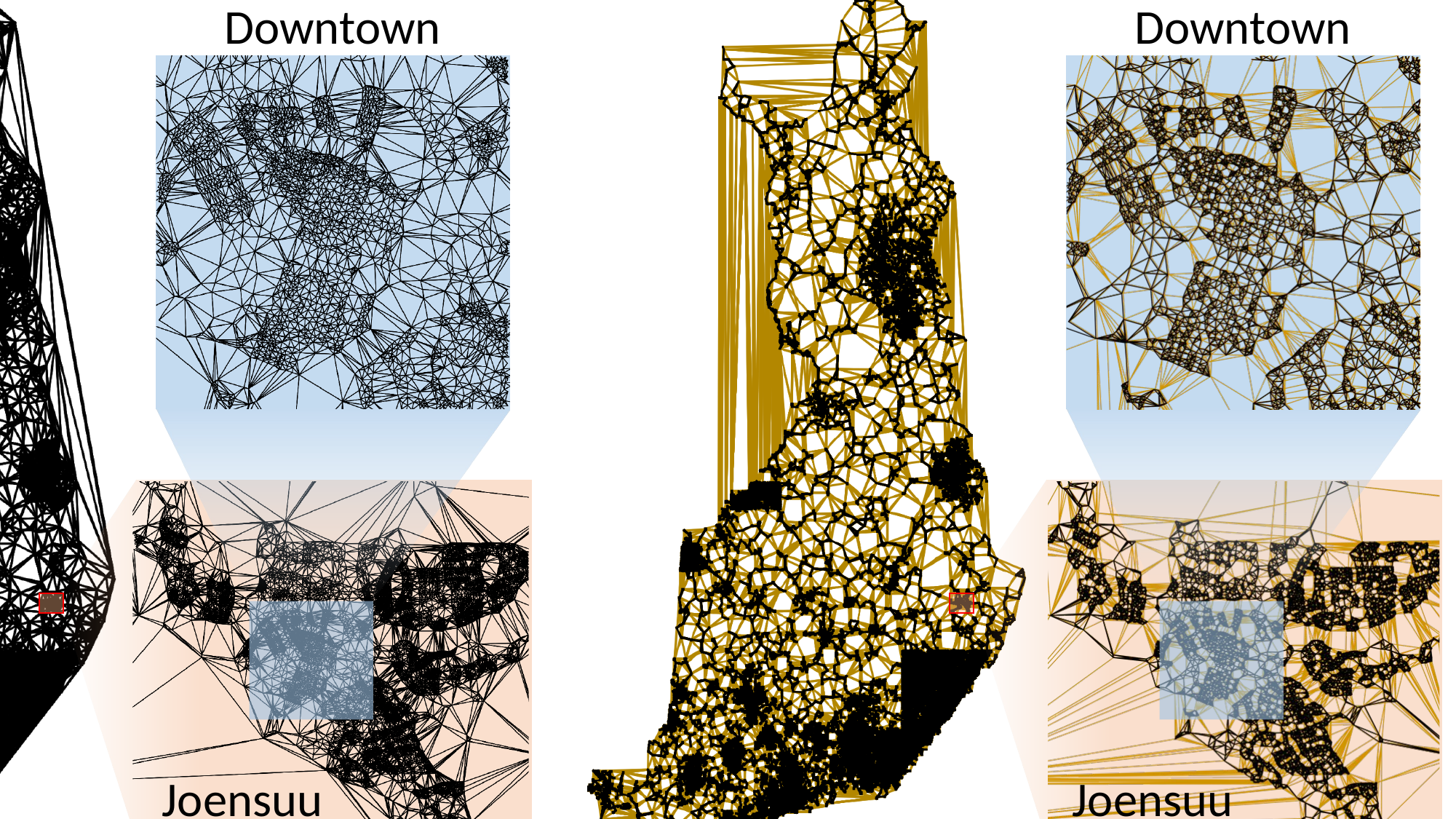

Delaunay Graph
5 Delaunay & 4 Quadrant
Downtown
Downtown
Joensuu
Joensuu

## Slide 10
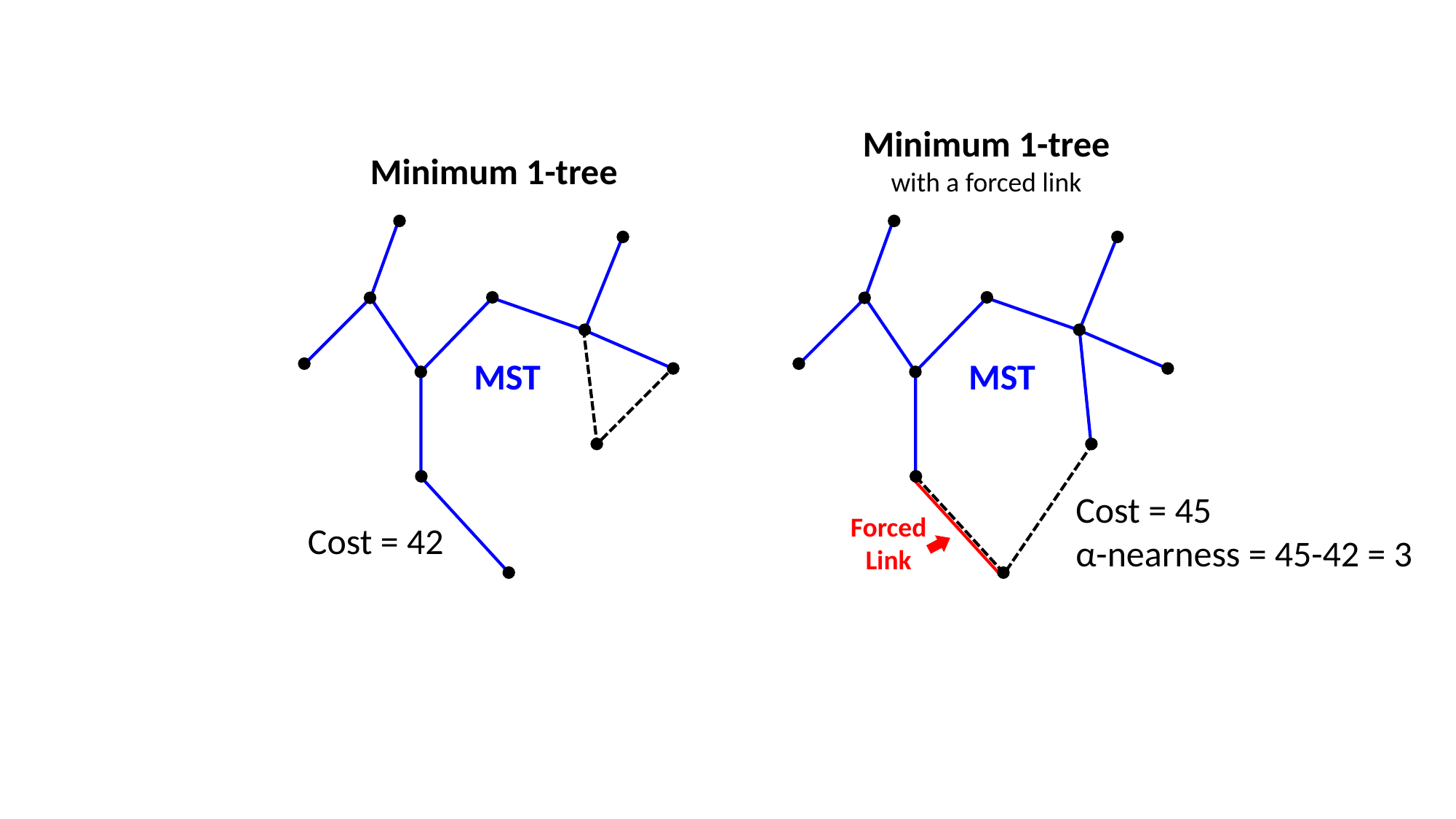

Minimum 1-tree
with a forced link
Minimum 1-tree
MST
MST
Cost = 45
α-nearness = 45-42 = 3
Forced
Link
Cost = 42

## Slide 11
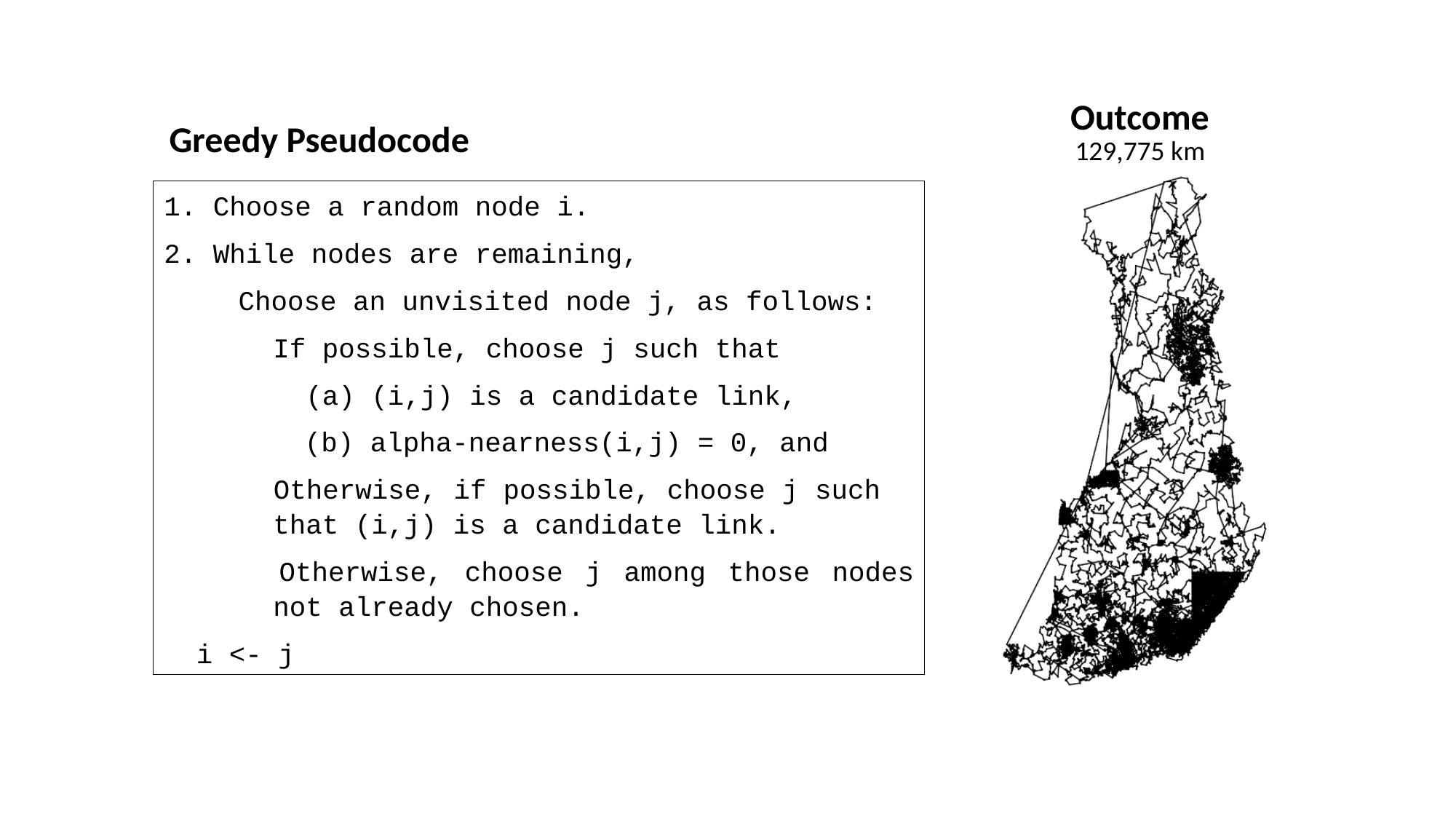

Outcome
Greedy Pseudocode
129,775 km
1. Choose a random node i.
2. While nodes are remaining,
	Choose an unvisited node j, as follows:
 		If possible, choose j such that
 		 (a) (i,j) is a candidate link,
 	 (b) alpha-nearness(i,j) = 0, and
 		Otherwise, if possible, choose j such 		that (i,j) is a candidate link.
 		Otherwise, choose j among those nodes 		not already chosen.
	i <- j

## Slide 12
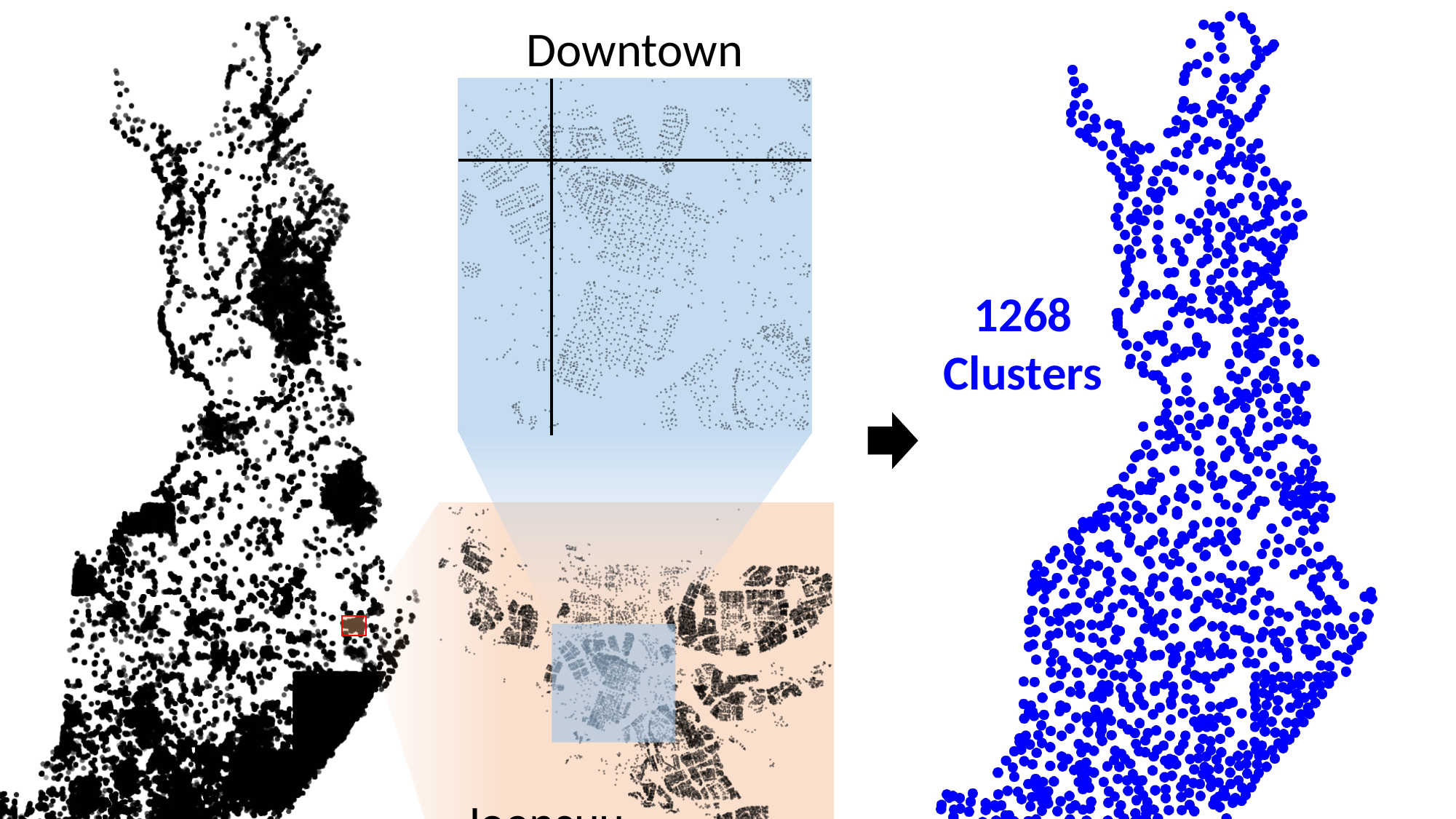

Downtown
Joensuu
1268
Clusters

## Slide 13
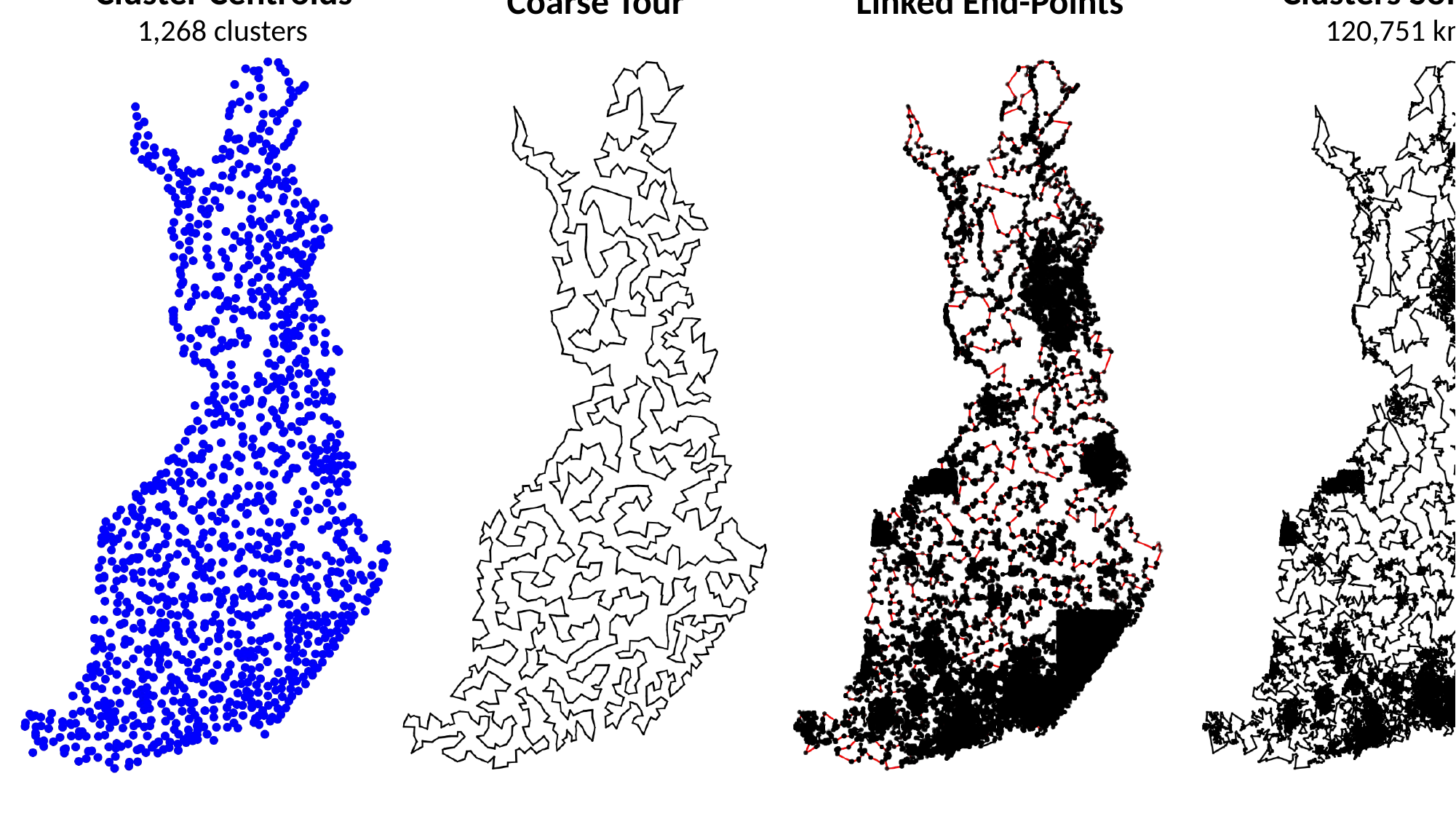

Cluster Centroids
Clusters Solved
Coarse Tour
Linked End-Points
1,268 clusters
120,751 km

## Slide 14
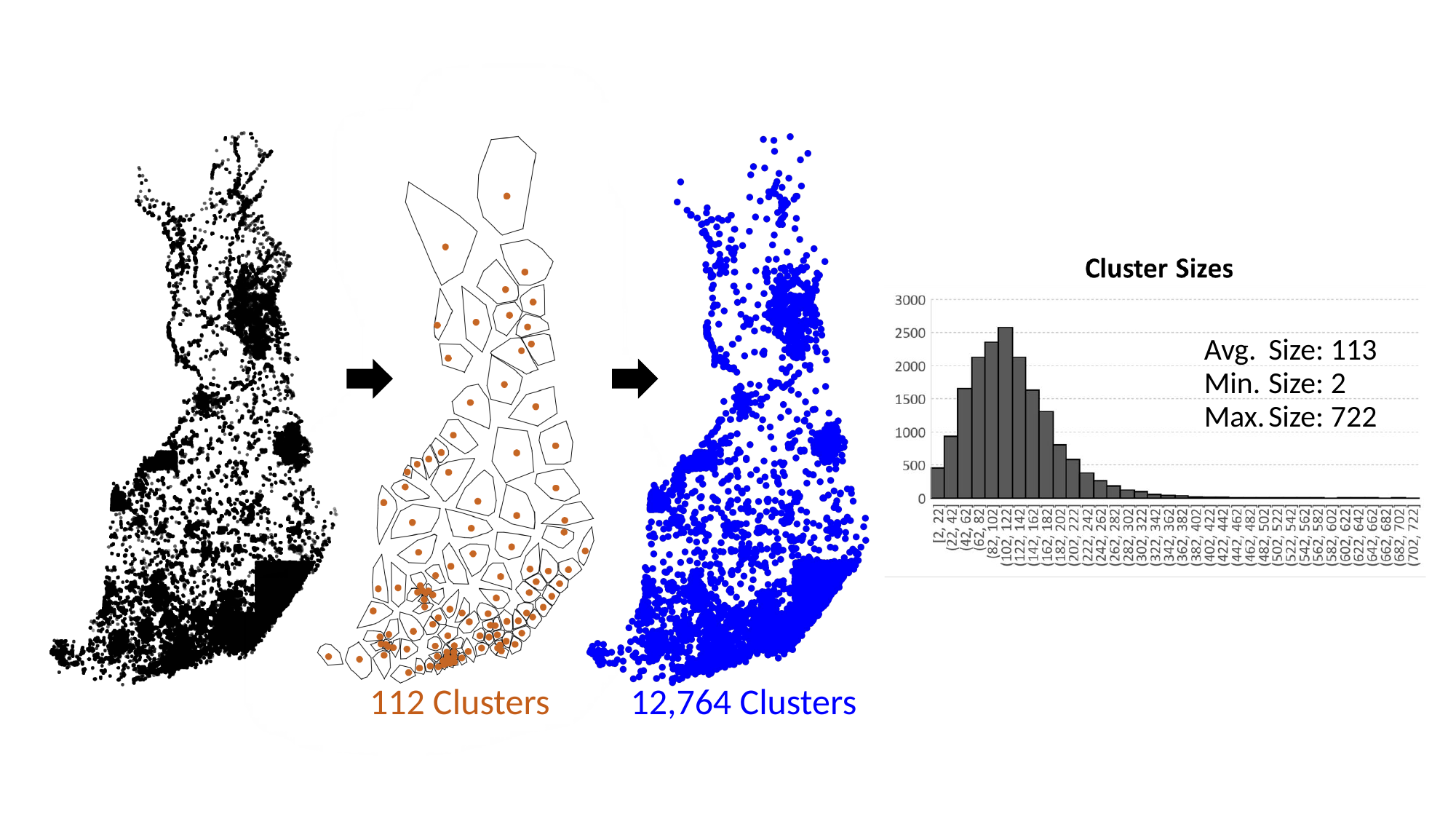

Avg. 	Size: 113
Min. 	Size: 2
Max.	Size: 722
112 Clusters
12,764 Clusters

## Slide 15
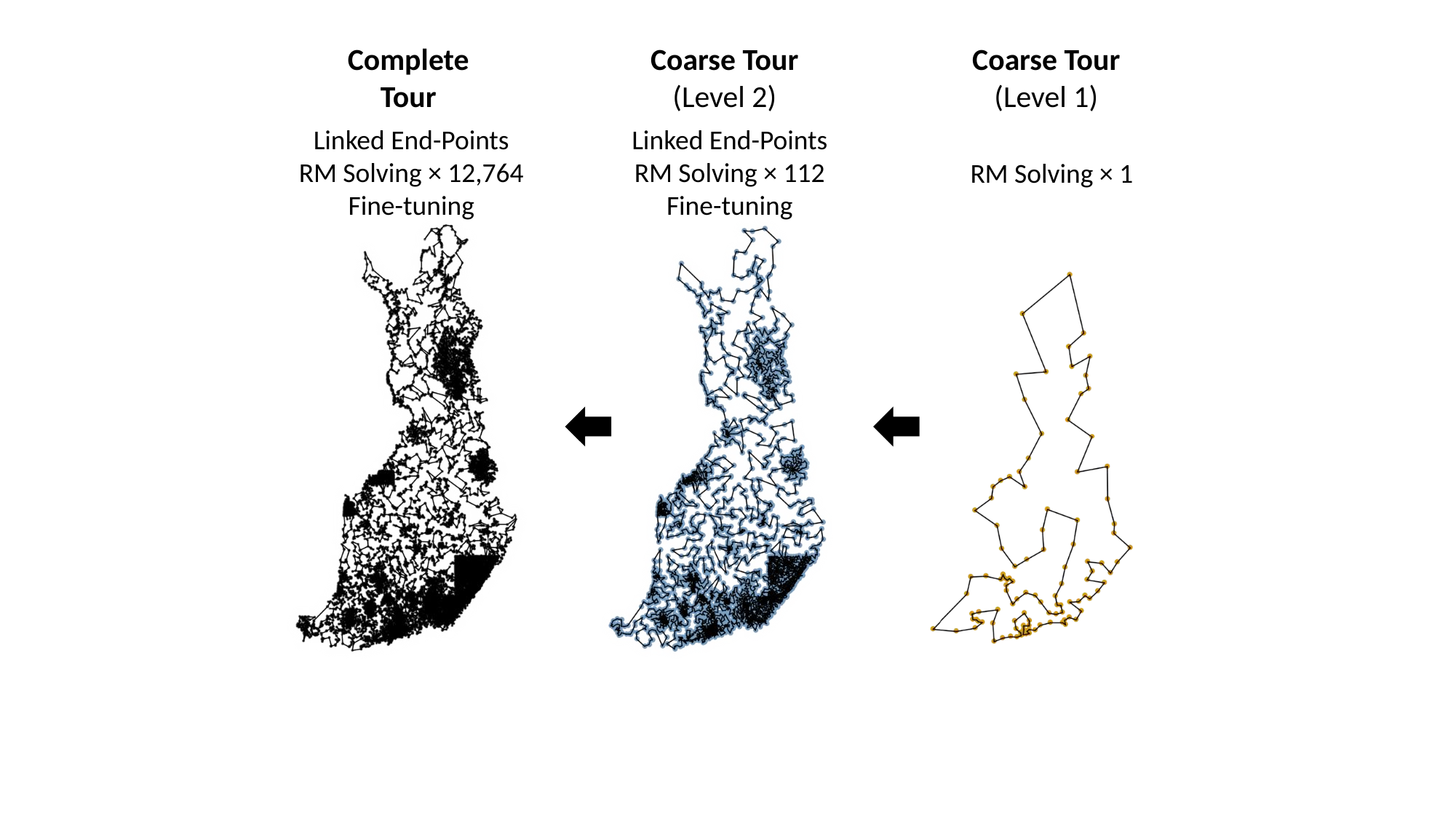

Complete
Tour
Coarse Tour
(Level 2)
Coarse Tour
(Level 1)
Linked End-Points
RM Solving × 12,764
Fine-tuning
Linked End-Points
RM Solving × 112
Fine-tuning
RM Solving × 1

## Slide 16
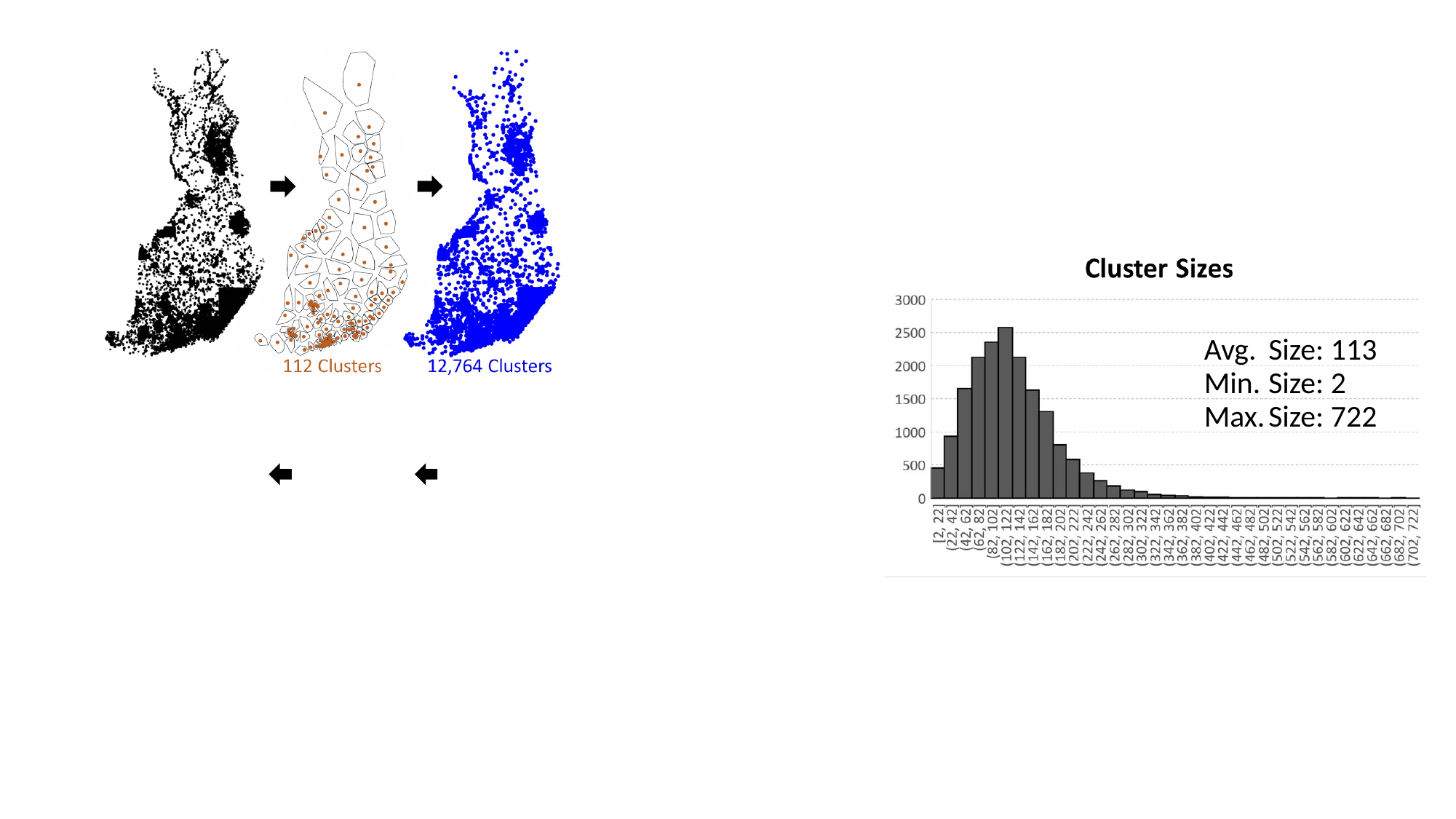

Avg. 	Size: 113
Min. 	Size: 2
Max.	Size: 722

## Slide 17
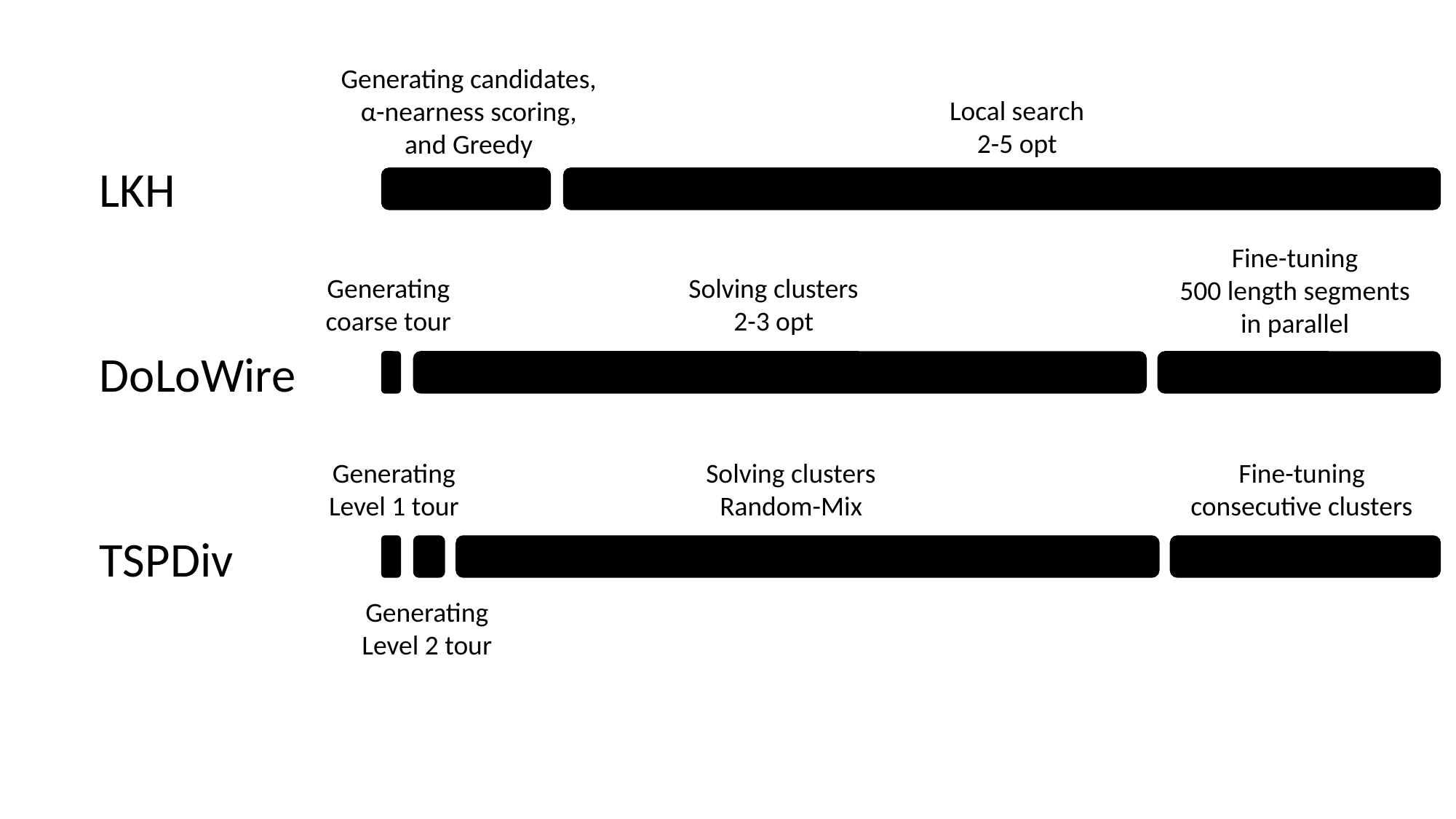

Generating candidates,
α-nearness scoring,
and Greedy
Local search
2-5 opt
LKH
Fine-tuning
500 length segments
in parallel
Generating
coarse tour
Solving clusters
2-3 opt
DoLoWire
Generating
Level 1 tour
Solving clusters
Random-Mix
Fine-tuning
consecutive clusters
TSPDiv
Generating
Level 2 tour

## Slide 18
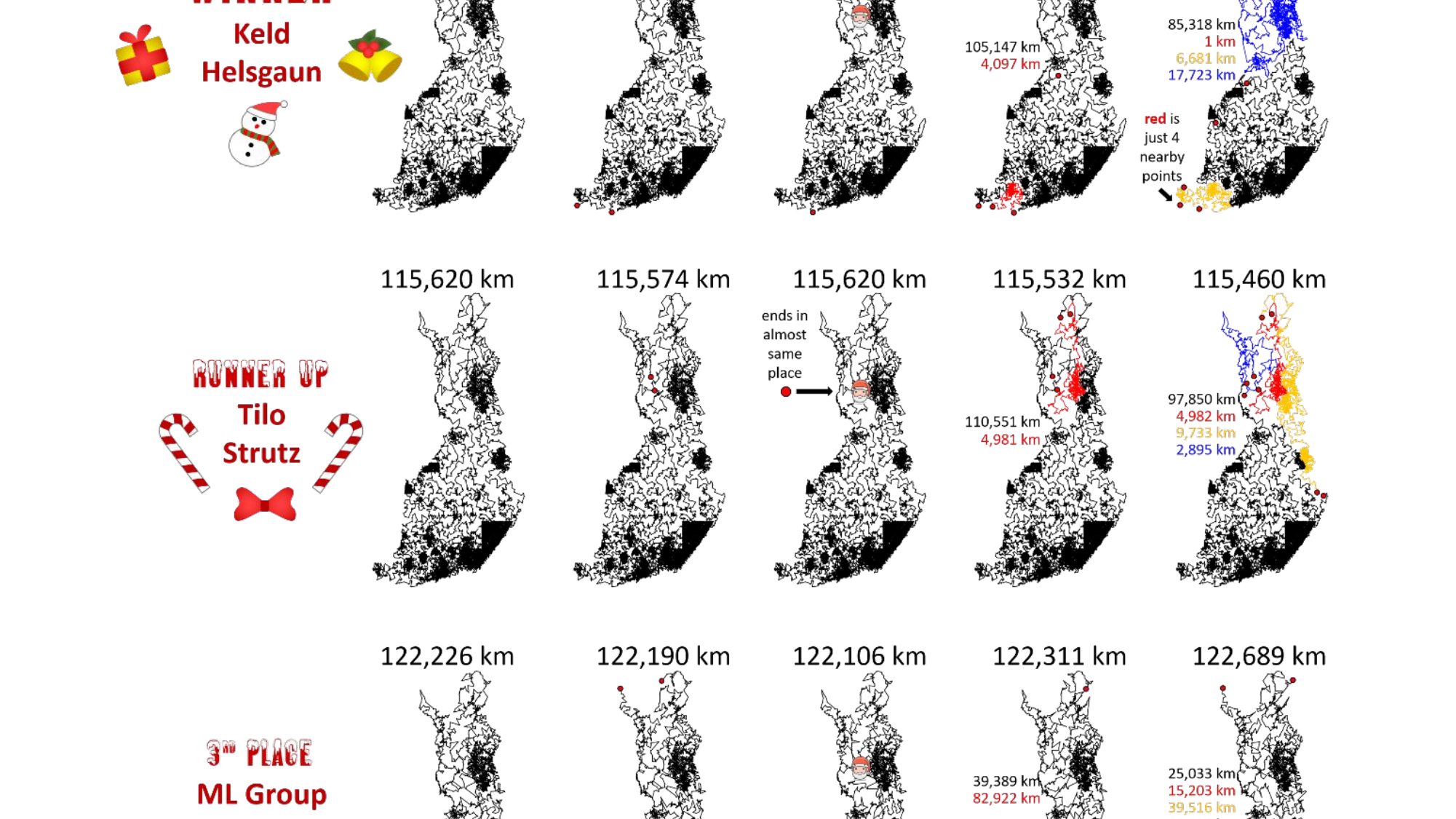

## Slide 19
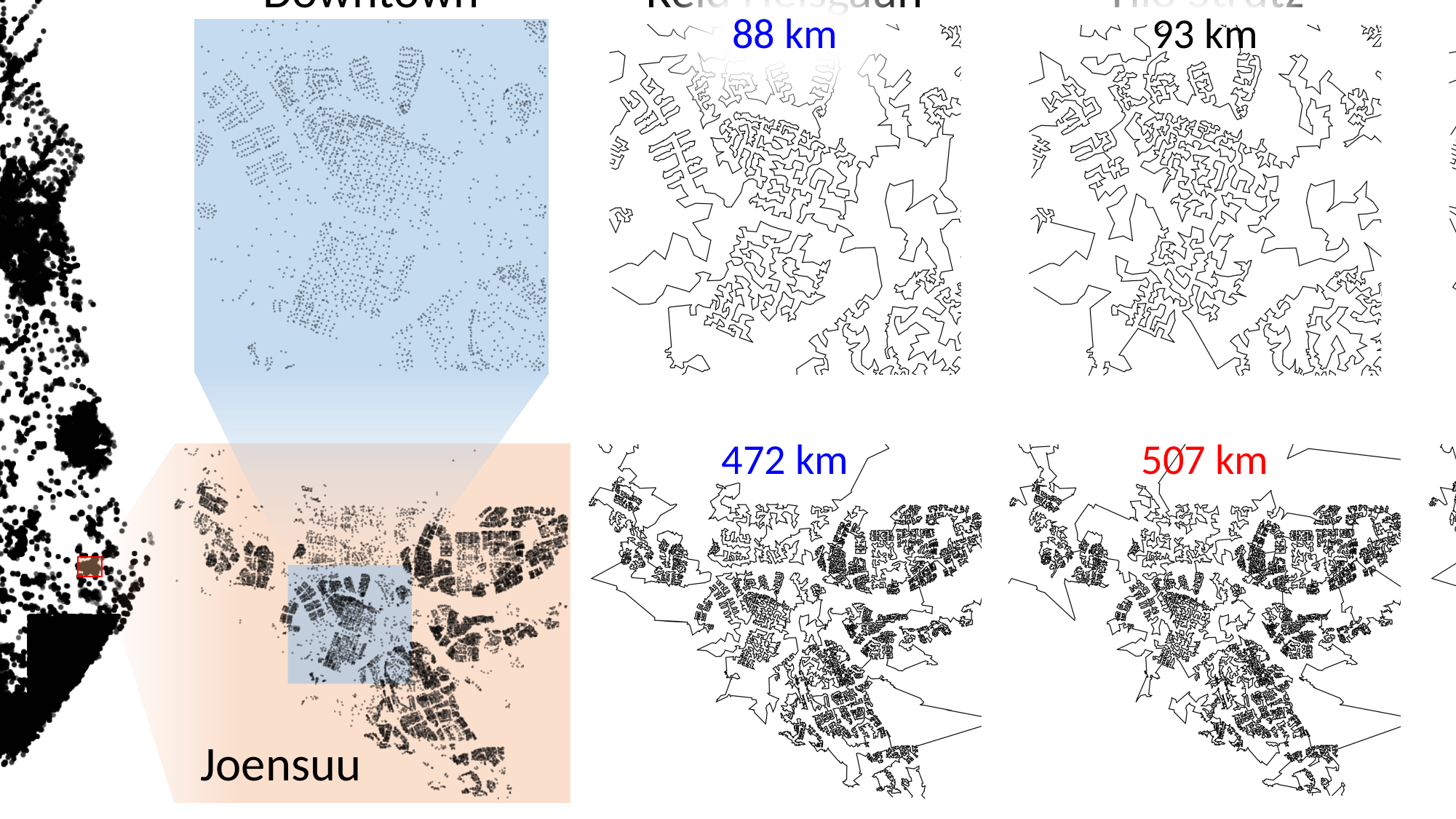

Downtown
Keld Helsgaun
Tilo Strutz
TSPDiv
88 km
93 km
95 km
472 km
507 km
506 km
Joensuu

## Slide 20
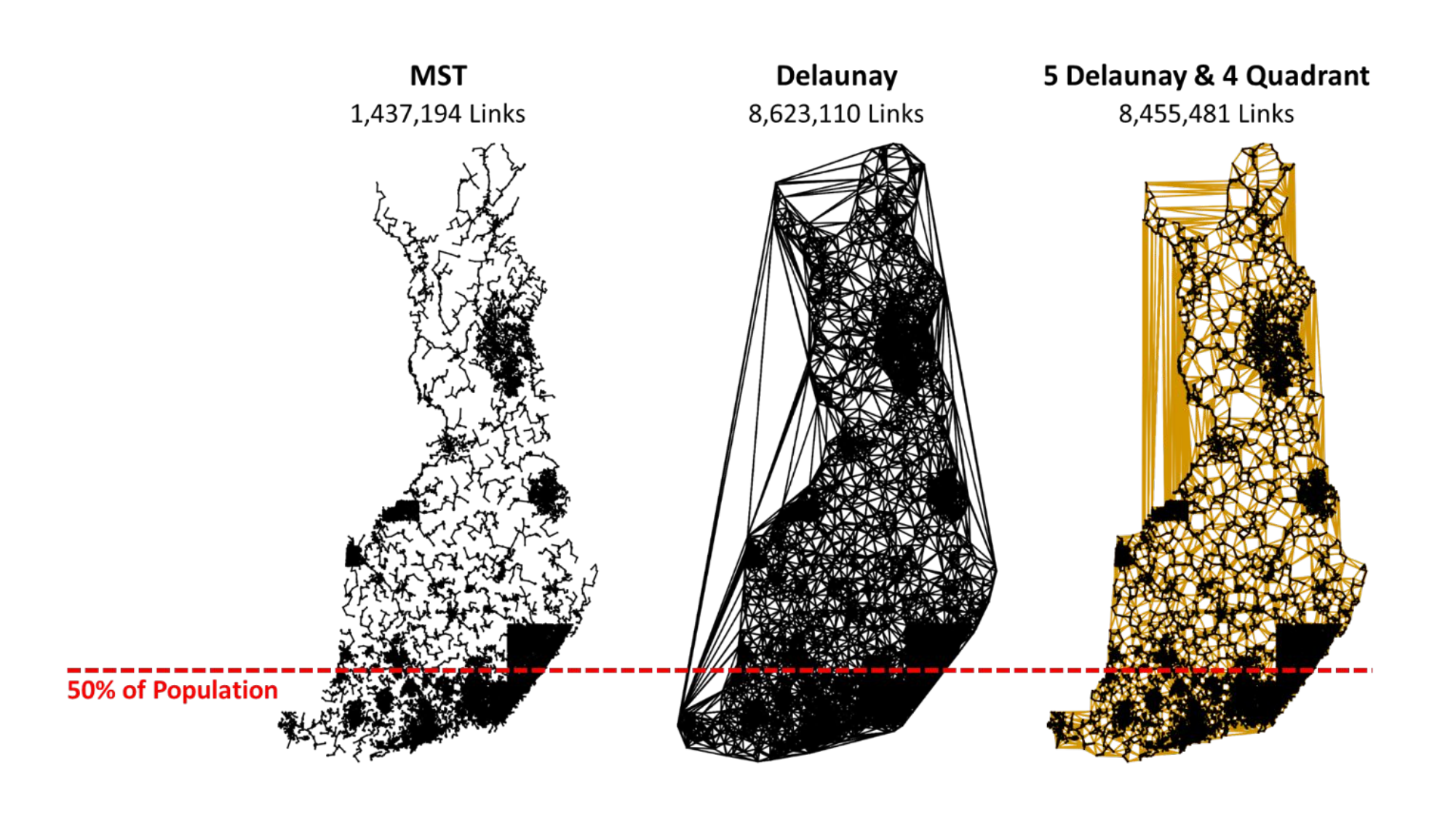

## Slide 21
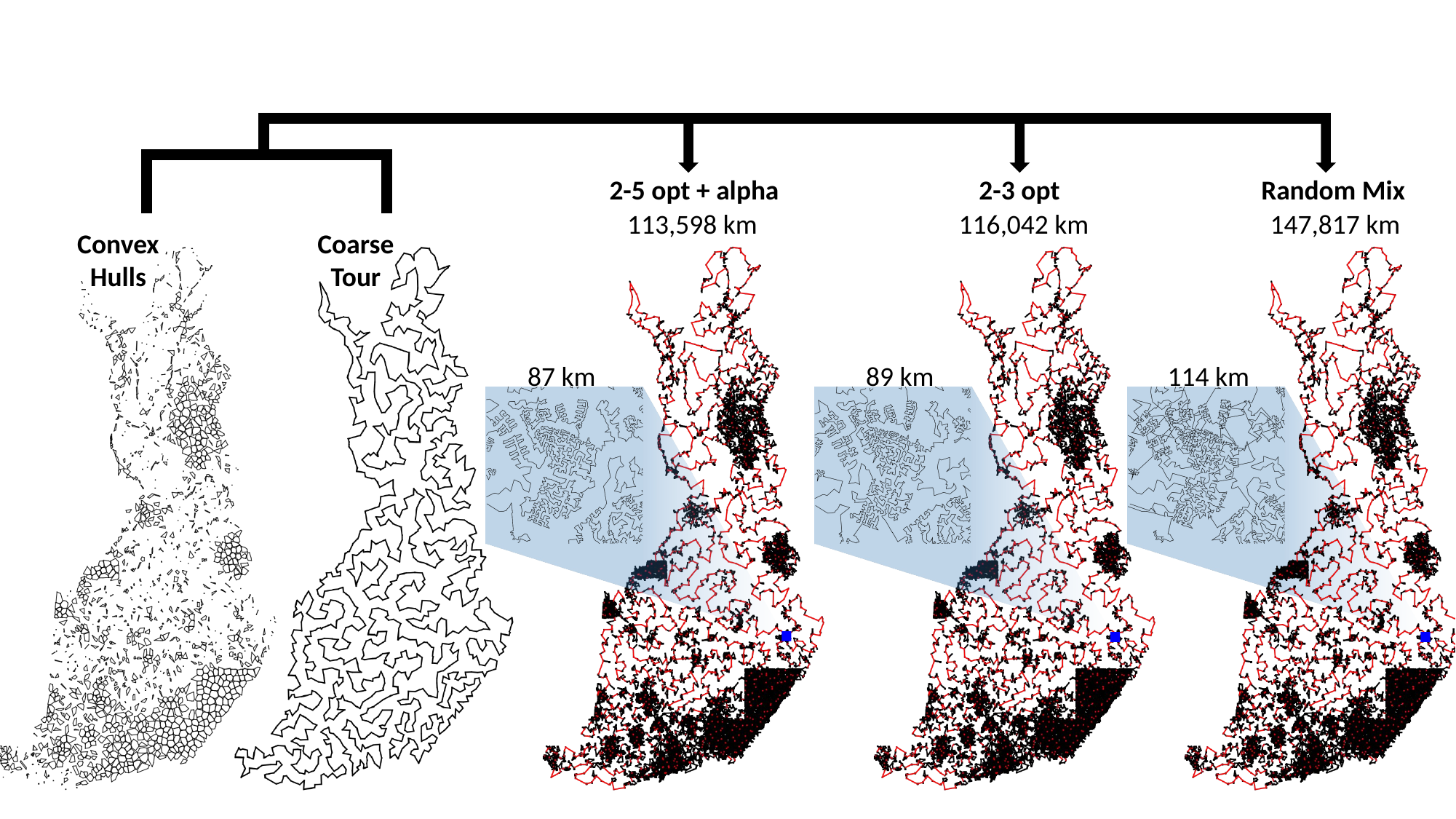

2-5 opt + alpha
2-3 opt
Random Mix
113,598 km
116,042 km
147,817 km
Convex
Hulls
Coarse
Tour
87 km
89 km
114 km

## Slide 22
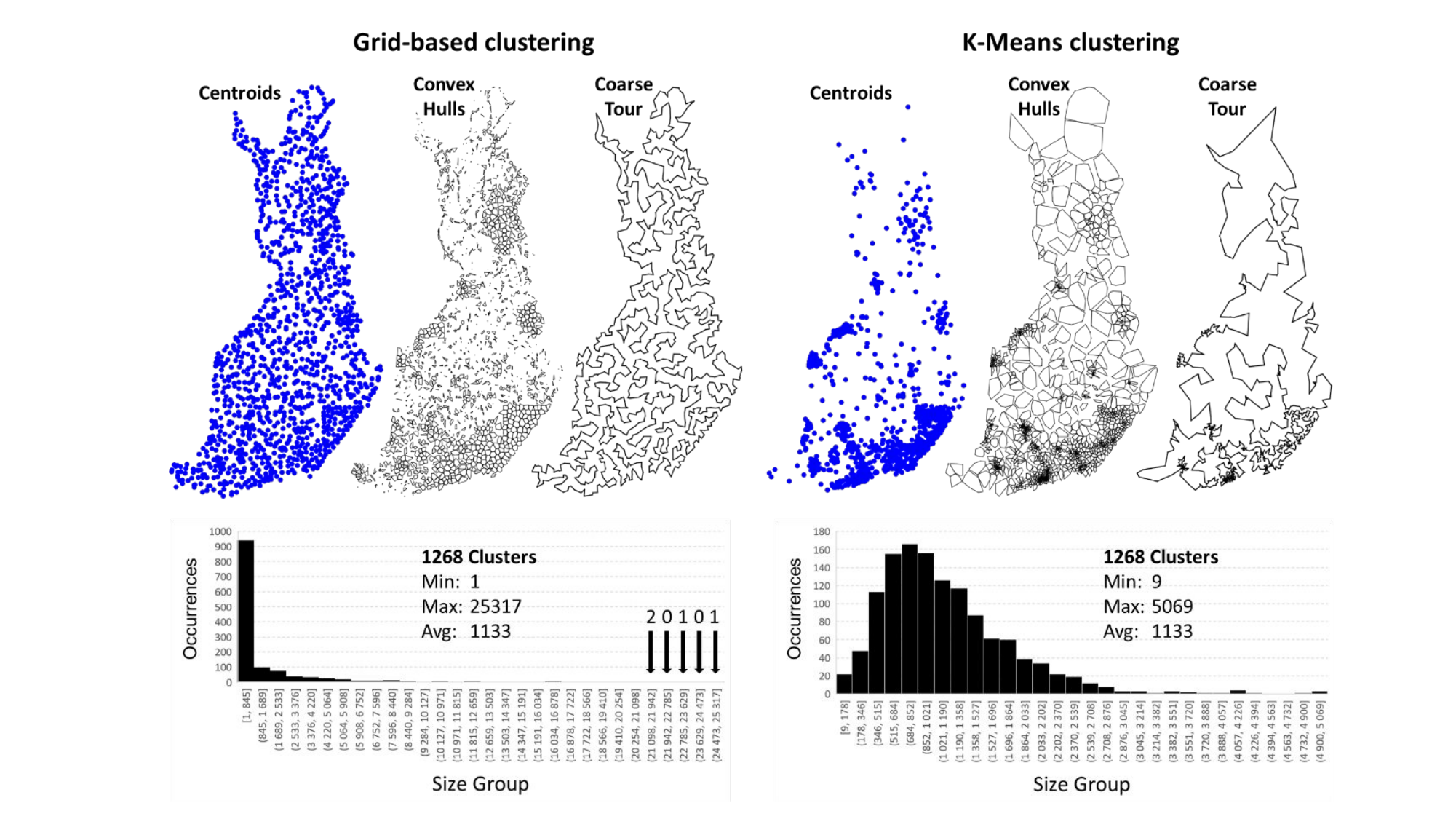

## Slide 23
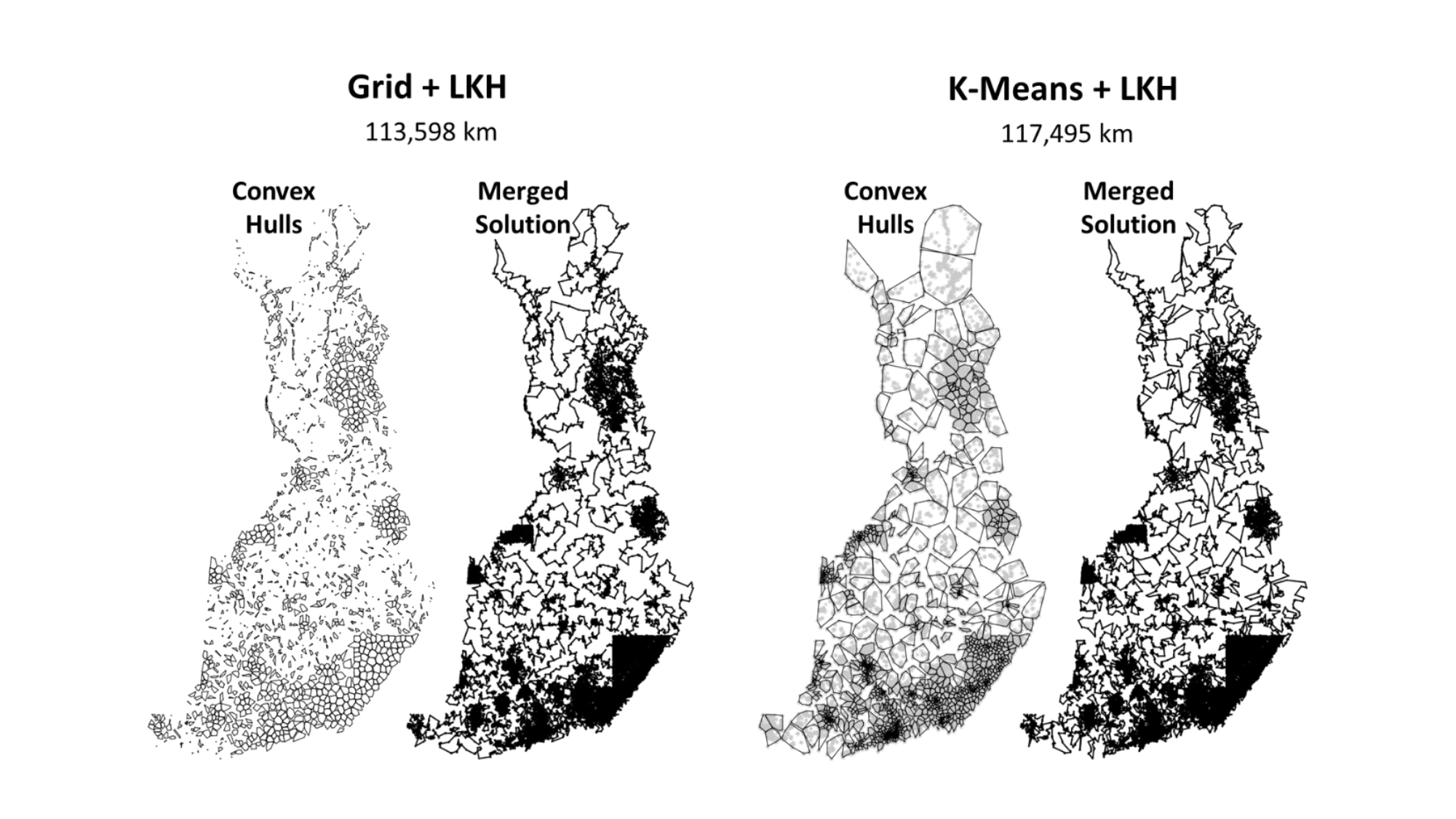

## Slide 24
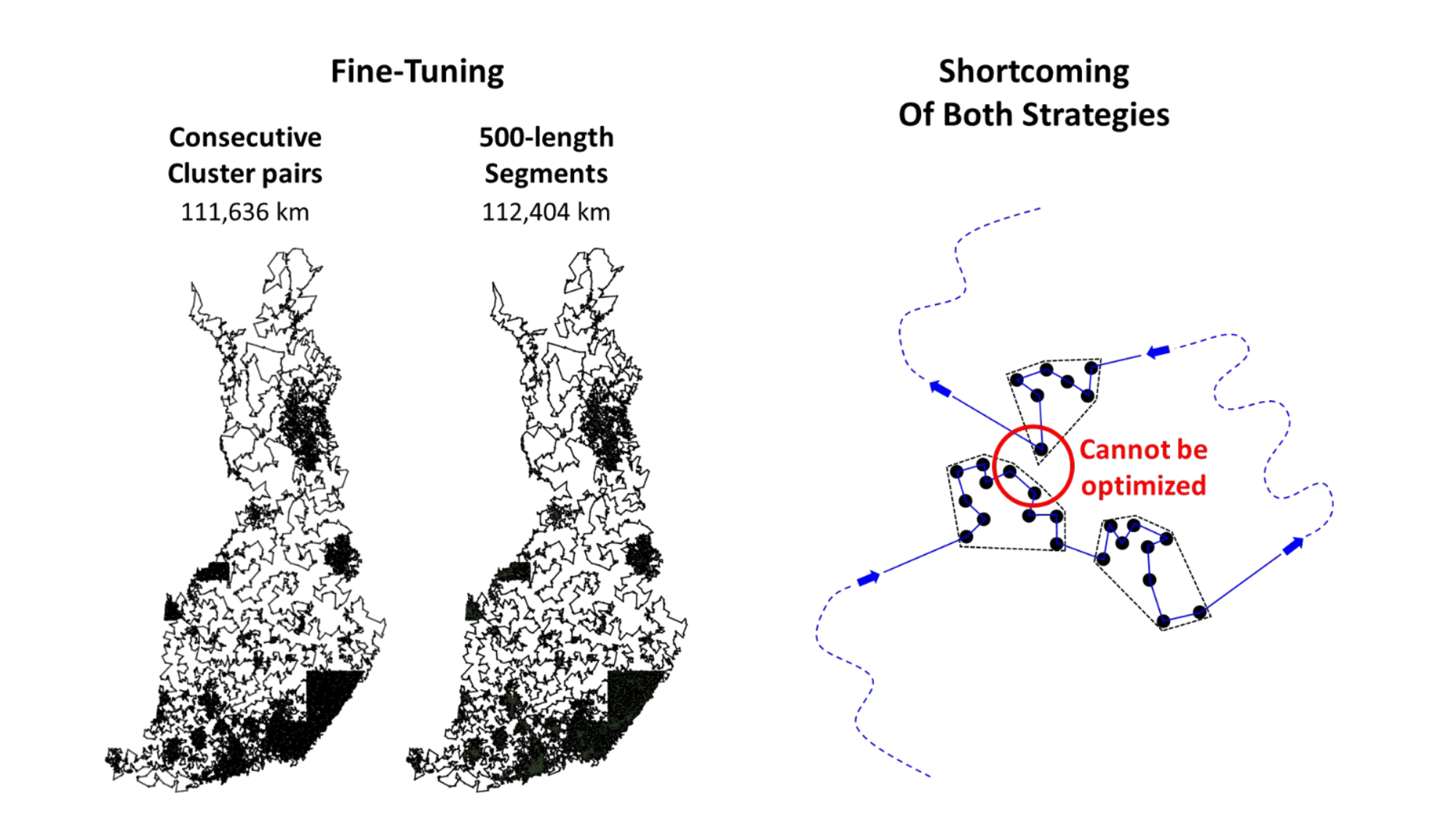

Supplement: Supplementary file 1 [file Presentation1.pptx]
